# Stable Gene Targeting in Human Cells Using Single-Strand Oligonucleotides with Modified Bases

**DOI:** 10.1371/journal.pone.0036697

**Published:** 2012-05-14

**Authors:** Xavier Rios, Adrian W. Briggs, Danos Christodoulou, Josh M. Gorham, Jonathan G. Seidman, George M. Church

**Affiliations:** Department of Genetics, Harvard Medical School, Boston, Massachusetts, United States of America; Saint Louis University, United States of America

## Abstract

Recent advances allow multiplexed genome engineering in *E. coli*, employing easily designed oligonucleotides to edit multiple loci simultaneously. A similar technology in human cells would greatly expedite functional genomics, both by enhancing our ability to test how individual variants such as single nucleotide polymorphisms (SNPs) are related to specific phenotypes, and potentially allowing simultaneous mutation of multiple loci. However, oligo-mediated targeting of human cells is currently limited by low targeting efficiencies and low survival of modified cells. Using a HeLa-based *EGFP*-rescue reporter system we show that use of modified base analogs can increase targeting efficiency, in part by avoiding the mismatch repair machinery. We investigate the effects of oligonucleotide toxicity and find a strong correlation between the number of phosphorothioate bonds and toxicity. Stably *EGFP*-corrected cells were generated at a frequency of ~0.05% with an optimized oligonucleotide design combining modified bases and reduced number of phosphorothioate bonds. We provide evidence from comparative RNA-seq analysis suggesting cellular immunity induced by the oligonucleotides might contribute to the low viability of oligo-corrected cells. Further optimization of this method should allow rapid and scalable genome engineering in human cells.

## Introduction

New sequencing technologies are producing a wealth of data, and soon it will be possible to sequence full human genomes for about $1000 [1]–[2]. Unfortunately, our current ability to sequence and identify genetic variants greatly outpaces our ability to characterize their functional consequences. Genome-wide association studies (GWAS) have revealed many single-nucleotide polymorphisms (SNPs) linked to disease phenotypes, most of which are ‘tag SNPs’ in the context of large genomic regions where the actual causal variants remain unknown [3]. As of this writing, NHGRI’s GWAS Catalog contains 6,030 SNPs with a p-value <1.0×10^−5^. Ultimately, potential causal variants linked to these SNPs need to be experimentally tested. Traditionally, targeted genetic modifications are achieved by selecting for rare homologous recombination(HR) events using a donor DNA that carries the desired mutation as well as a selectable gene marker; after selection, the marker may be removed using a targeted recombinase such as Cre. However, the throughput of this approach is limited by the extremely low frequency of HR(10^−5^–10^−7^) in human cells and the serial clonal isolation steps [4]. In addition, selection markers or residual recombinase sequences could confound subtle phenotypes such as those caused by variants in regulatory regions, which are of particular interest since expression variability of ~5% of all human genes is linked to SNPs located within 200 kb of the gene [5].

Custom targeted zinc-finger nucleases (ZFNs) [6], and more recently transcription activator-like effector nucleases(TALENs) [7] have shown great promise by increasing homologous recombination rates to up to ~20%, which allows screening of seamlessly-modified cells. ZFNs can be used with ~120 bp single-stranded oligonucleotides, which greatly simplifies donor DNA preparation [8]. However, production of custom nucleases for each new locus is dependent on knowledge of protein-DNA binding specificities, laborious design and optimization methods or expensive commercial licensing [9]–[11]. In addition, the potential for multiplexing custom targeted nucleases may be limited since multiple double strand breaks could lead to gross genome rearrangements or deletions [12]–[13]. Thus, a different genome engineering technology will be necessary to keep up with the demand for post-GWAS functional characterizations.

Oligonucleotide-mediated targeting is an alternative strategy for genome engineering [14], in which mutation-encoding oligonucleotides modify the genome without the need for custom-designed DNA-binding proteins. The inherent simplicity and scalability of this method makes it particularly well suited for generating a large number of genetic variants. In *E. coli* λ Red-mediated oligonucleotide recombineering, an oligo preferentially anneals to the lagging strand of the genome during DNA replication and incorporates into the daughter strand [15]. This has been optimized to achieve targeting efficiencies as high as 20%, and when performed with multiplexed degenerate oligos can generate >4×10^6^ combinatorial genomic variations [16]. A similar method has been developed in mammalian cells, with reported gene targeting frequencies of up to 2% [17]–[20]. There is strong evidence showing oligo incorporation [18] and strand bias [21]–[22]. Similarly to *E. coli,* the endogenous mismatch repair (MMR) machinery has been found to inhibit oligo incorporation in mammalian cells [20], [23]–[24], and oligos modified with phosphorothioate (PTO) bonds to prevent nuclease degradation have been found to increase targeting efficiencies [20].

The main limitation of oligonucleotide-mediated targeting in mammalian cells is the low survival of modified cells, with only a few studies having demonstrated long-term survival and proliferation of corrected clones. To increase survival, the use of unprotected oligos has been suggested [25], [18], however this also makes corrections too low for multiplexed genome engineering. Here we describe our progress towards surpassing these limitations. Using an *EGFP*-rescue based reporter system in HeLa cells [19], we find that the use of commercially available chemically modified base analogs to encode the mismatch in the targeting oligo increase its frequency of incorporation, similar to results in *E. coli* [26]. We also find that the number of PTO modifications correlate with both the toxicity of oligo transfection and the proliferation of oligo-modified cells, and having 3–5 PTO bonds 3′ to the mismatch produced a >10-fold increase in stable targeting compared to an unmodified oligos. Applying these oligo design principles we were able to stably correct a mutant *EGFP* with an efficiency of ~0.05% in HeLa cells. Finally, we compared the transcription profiles of *EGFP-*corrected and -uncorrected cells via RNA-seq and find upregulation of many genes associated with cellular immunity. We propose a new model explaining the toxicity in oligo-mediated recombination in human cells, where cells that uptake more oligo are more likely to become corrected, and likewise these cells are more likely to activate cellular immune responses. Future exploration of this cellular response to transfected oligos may lead to novel strategies for reducing their toxicity and eventually enable multiplexed genome engineering in human cells.

## Results

### Mismatch-specific *EGFP* Correction

**Figure 1:**
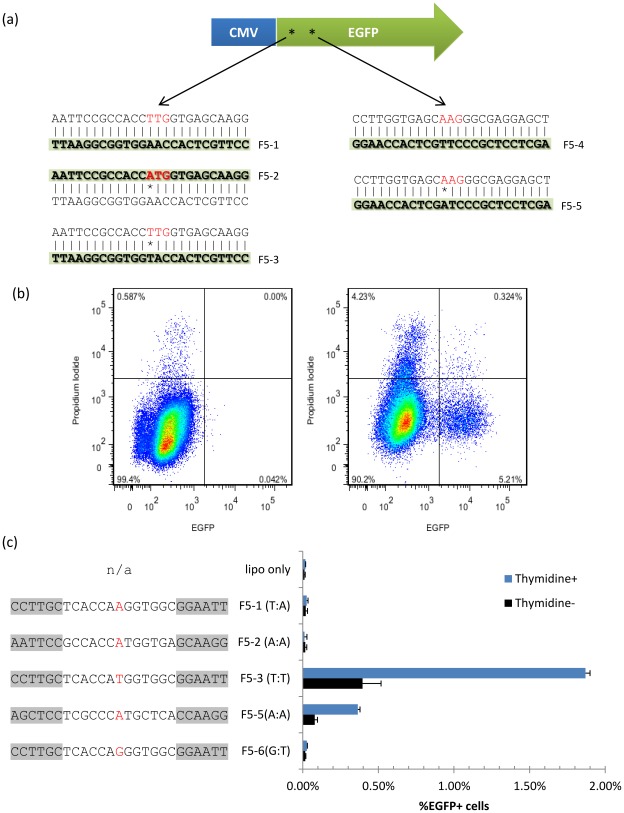
Oligo-mediated targeting reporter system. (a) Reporter consists of a HeLa cell line with two stably integrated copies of EGFP with a mutated TTG start codon and a second potential start codon (AAG) downstream, both shown in red. Each mutated start codon can be targeted by sense or antisense oligos. Representative oligos used in this study are highlighted, in duplex form, with the mismatch shown by an asterisk. Further detail can be found on Table 1. (b) Sample flow cytometry dot plot of cells with no oligo (left) or transfected with F5-8 (right, Table 1). The frequency of oligo-induced correction of a start codon can be estimated as the %EGFP+/Propidium Iodide- cells (c) %EGFP+ cells generated after different oligo transfections, with and without 20 mM thymidine, assayed 36 h post transfections. Oligo sequences are shown 5′ to 3′, and the PTO bonds are highlighted in gray. The control oligos F5-1 and F5-6 did not produce any significant proportion of EGFP+ cells, neither did F5-2, which is complementary to the transcribed strand and encodes an ATG-restoring mutation. The oligos targeting the first and second potential start codons on the non-transcribed strand, F5-3 and F5-5 respectively, did produce EGFP+ cells. Lipo = lipofectamine only control. n = 4.

To improve human genome engineering with oligonucleotides, we worked with a well-characterized reporter system [19] consisting of a HeLa cell line with two stably integrated copies of a modified *EGFP* gene (*mEGFP*). This version of *EGFP* has a non-functional start codon (TTG) which can be rescued by targeting oligos (Figure 1a, Table 1) encoding for a functional ATG, thus the oligo-mediated targeting efficiency can be determined by flow cytometry as the percentage EGFP+ cells (Fig. 1b). We confirmed previous findings [27] where targeting *mEGFP* with a 25 bp long oligo complementary to the non-transcribed strand and carrying a centrally located mismatch and six PTO bonds at each end (F5-3, Fig. 1c) delivered with Lipofectamine 2000 yielded a substantial proportion of EGFP+ cells after 48 hours (~0.5%), and this efficiency was further increased to ~2% by slowing down DNA replication with thymidine treatment. Other cationic lipid transfection reagents resulted in lower efficiencies, possibly due to low nuclear accumulation of the oligo (Figures S1,S2). Alternative delivery methods such as electroporation and nucleofection failed to produce any significant proportion of EGFP+ cells (not shown). An oligo complementary to the transcribed strand, F5-2, did not produce any EGFP+ cells significantly different from background. Transfection of an oligo encoding an alternative ATG-restoring mutation 9 bp away from the first site resulted in ~0.4% *EGFP*+ cells (F5-5, Fig. 1a,c), whereas an oligo carrying a non-coding mismatch (F5-6, Fig. 1c) did not produce EGFP+ cells, demonstrating that the expression of EGFP depends on the targeting oligo sequence restoring the ORF. To further verify that EFGP+ cells had undergone the desired genomic modification, EGFP+ and – cells were sorted out by fluorescent-activated cell sorting (FACS) and genotyped by allele-specific qPCR (AS-qPCR). The EGFP+ population was estimated by AS-qPCR to carry 11–13% converted DNA, which matches the 12.5% expected if these cells have undergone a single oligo incorporation at one of the two genomic *mEGFP* during DNA replication (1/8 strands at the end of S phase) but not yet proceeded through cell division. In the EGFP- population, the proportion of corrected alleles was estimated to be ~1%, which may either represent corrected cells that had not yet produced functional EGFP protein or PCR artifacts caused by residual targeting oligonucleotides in the cells [28].

**Table 1:** Oligonucleotides used in this study.

Oligo name	Sequence	Corresponding Fig. 1 strand
F5-1	C*C*T*T*G*C*TCACCAAGGTGGC*G*G*A*A*T*T	F5-1
F5-2	A*A*T*T*C*C*GCCACCATGGTGA*G*C*A*A*G*G	F5-2
F5-3	C*C*T*T*G*C*TCACCATGGTGGC*G*G*A*A*T*T	F5-3
F5-4	A*G*C*T*C*C*TCGCCCTTGCTCA*C*C*A*a*G*G	F5-4
F5-5	A*G*C*T*C*C*TCGCCCATGCTCA*C*C*A*A*G*G	F5-5
F5-6	A*G*C*T*C*C*TCGCCCGTGCTCA*C*C*A*A*G*G	F5-3
F5-7	C*C*T*T*G*C*TCACCAdUGGTGGC*G*G*A*A*T*T	F5-3
F5-8	C*C*T*T*G*C*TCACCAFuGGTGGC*G*G*A*A*T*T	F5-3
F5-9	A*G*C*T*C*C*TCGCCCFaTGCTCA*C*C*A*A*G*G	F5-4
F5-10	A*G*C*T*C*C*TCGCCCAmTGCTCA*C*C*A*A*G*G	F5-4
F5-11	C*C*T*T*G*C*TCACCATGGTGGCGGAATT	F5-3
F5-12	CCTTGCTCACCATGGTGGC*G*G*A*A*T*T	F5-3
F5-13	C*C*T*TGCTCACCATGGTGGCGGA*A*T*T	F5-3
F5-14	C*C*T*T*G*CTCACCATGGTGGCG*G*A*A*T*T	F5-3
F5-15	C*C*T*T*G*C*T*CACCATGGTGG*C*G*G*A*A*T*T	F5-3
F5-16	C*C*T*T*G*C*T*C*A*CCATGGT*G*G*C*G*G*A*A*T*T	F5-3
F5-17	CCTTGCTCACCAFu*G*G*TGGCGGAATT	F5-3
F5-18	CCTTGCTCACCAFu*G*G*T*G*G*CGGAATT	F5-3
F5-19	C*C*T*T*G*C*TCACCAFuGGTGGC*G*G*A*A*T*T	F5-3
F5-20	CCTTGCTCACCAA*G*G*TGGCGGAATT	F5-1
F5-21	CCTTGCTCACCAFuGGTGGCGGAATT	F5-3
F5-22	CCTTGCTCACCAFu*GGTGGCGGAATT	F5-3
F5-23	CCTTGCTCACCAFu*G*GTGGCGGAATT	F5-3
F5-24	CCTTGCTCACCAFu*G*G*T*GGCGGAATT	F5-3
F5-25	CCTTGCTCACCAFu*G*G*T*G*GCGGAATT	F5-3
F5-26	CCTTGCTCACCAFu*G*G*T*G*G*C*GGAATT	F5-3
F5-27	CCTTGCTCACCAFu*G*G*T*G*G*C*G*GAATT	F5-3
F5-28	CCTTGCTCACCAFu*G*G*T*G*G*C*G*G*AATT	F5-3
F5-29	CCTTGCTCACCAT*G*G*TGGCGGAATT	F5-3
F5-30	CCTTGCTCACCAFuGGTGGCGGA*A*T*T	F5-3
F5-31	AATTCCGCCACCA*T*G*GTGAGCAAGG	F5-2
F5-32	AATTCCGCCACCAm*T*G*GTGAGCAAGG	F5-2
F5-33	CCTTGCTCACCAFu*A*G*TGGCGGAATT	F5-3
F5-34	CCTTGCTCACCAFu*A*G*GTGGCGGAAT	F5-3
F5-35	CCTTGCTCACCAT*A*G*TGGCGGAATT	F5-3
F5-36	CCTTGCTCACCAT*A*G*GTGGCGGAAT	F5-3
F5-37	Cy5-GGCTCCTTCAGCT*G*T*GAGACTACGT	n/a
F5-38	CCTTGCTCACCAFu*G*G*TGGmCGGAATT	F5-3

Sequences shown 5′ to 3′. PTO bonds shown as asterisks (*). dU = deoxyUridine, Fu = 2′-Fluorouracil, Fa = 2′-Fluoroadenine, Am = 2-Aminopurine, mC = 5-Methyl deoxyCytidine.

### Modified Bases Increase Efficiency in Part by Avoiding Mismatch Repair in Mammalian Cells

**Figure 2:**
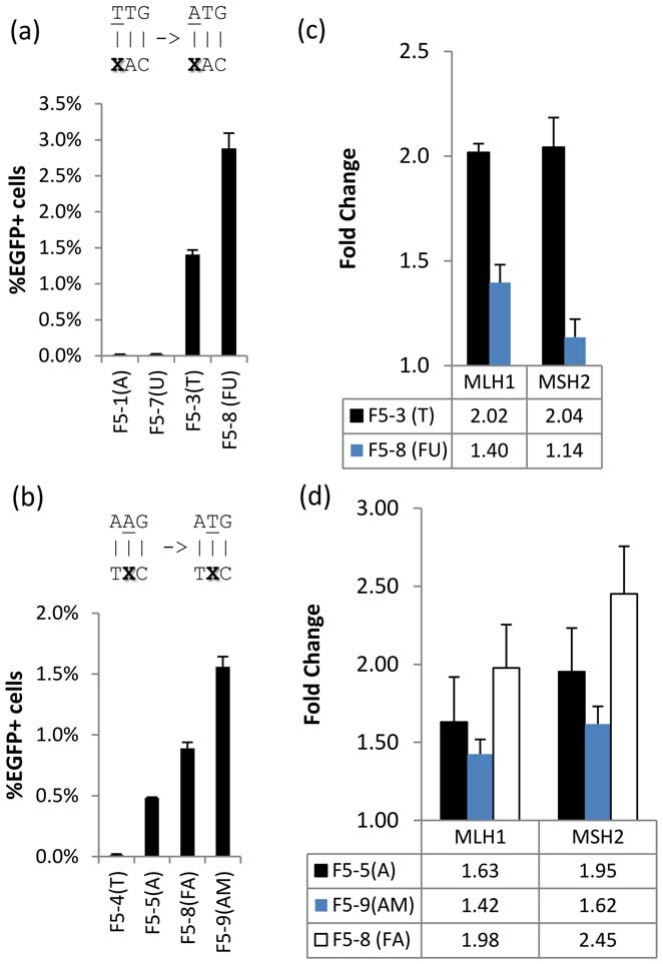
Chemically-modified base analogs. (a–b)Modified base-containing oligos complementary to the non-transcribed strand’s (a)first potential start codon TTG and (b) second potential start codon AAG, where each mismatched base X in the targeting oligo is shown in parenthesis. (c–d) RNAi targeting key mismatch repair proteins MLH1 and MSH2 for the (a)TTG and (b)AAG start codon targeted by oligos containing modified bases. Data was normalized relative to scr shRNA, thus in (c) each MMR component silencing produced a 2-fold improvement for the natural T base, while the improvement for FU was reduced. This is seen to a lesser degree in (d) comparing A and AM, while FA was further improved, suggesting it is more strongly recognized by MMR. n = 4.

Previous work has shown that the MMR machinery plays a significant role recognizing and removing the mutation caused by the oligo incorporation event. Currently, the only way around this is by either completely knocking down one of the main MMR proteins (e.g. MSH2) or by transient silencing with RNAi [29]. Our group recently showed an alternative strategy in *E. coli,* where the oligo contains chemically modified bases that avoid mismatch repair recognition. We tested oligos complementary to the non-transcribed strands of the two potential start codons of *mEGFP* while varying the mismatched base (Fig. 1a, Fig. 2). The best replacement for the T-T mismatch on the first start codon was 2′-Fluorouracil (FU), while for the A-A mismatch on the second start codon 2-Aminopurine (AM) was best, each giving a ~2-fold increase in *mEGFP* correction efficiency (Fig. 2a,b). To test whether the increased efficiency in *mEGFP* correction by the modified bases was due to avoidance of MMR, we transfected cells with validated shRNAs plasmids targeting *MSH2* and *MLH1*(Table S2), then tested the *mEGFP* correction efficiency using oligos with the different modified bases. Downregulating either MSH2 or MLH1,as confirmed by western blotting (Figure S3a), lead to a ~2-fold increase in *mEGFP* targeting efficiency in both the T-T and the A-A mismatched oligos (Fig. 2c,d). However, this increase was significantly lower for FU-T (Student t-test p-val = 1.26E-5 for *MLH1*, 3.42E-5 for *MSH2*) and somewhat lower for AM-A mismatch (p-val = 0.07 for *MLH1*). Interestingly, FA seems to be more strongly recognized by MMR than the natural base, suggesting an alternate mechanism is producing the increase in targeting efficiency observed. These results were verified by targeting MSH2 and MLH1 with siRNAs (Figure S3b,c). Thus, silencing *MSH2* and *MLH1* decreased the gap in targeting frequencies seen between the natural and modified bases, suggesting that the increase in targeting efficiency by oligos containing modified bases can be explained in part due to avoidance of MMR.

### Phosphorothioate Modifications Correlate with Oligo Transfection Toxicity

By integrating an oligo into the transcribed strand of *mEGFP* during DNA replication, oligo incorporation can be quantified early, since transcription and translation of *EGFP* can already take place before cell division. Aarts et. al. [25] showed that post-replication/pre-division corrected cells each contain only one corrected out of four strands at the individual target locus, so upon two rounds of cell division only 25% of cells stemming from the original EGFP+ population will still be *EGFP*+. However, we tracked the percentage of EGFP+ cells generated with oligo F5-3 over time and found an even greater decrease than expected; after 5 days EGFP+ had dropped to undetectable levels (not shown). This indicates that the corrected cells were either proliferating slower than the non-corrected cells or dying. To distinguish these possibilities we sorted corrected single cells 48 hrs post F5-3 oligo transfection into 96-well plates. After multiple attempts, no *EGFP*+ clones were detected even when using oligos with the FU base analog, suggesting that corrected cells were unable to proliferate. This is in agreement with the results from Liu et. al. [30], who were also unable to generate stably corrected clones with this HeLa F5 cell line.

**Figure 3:**
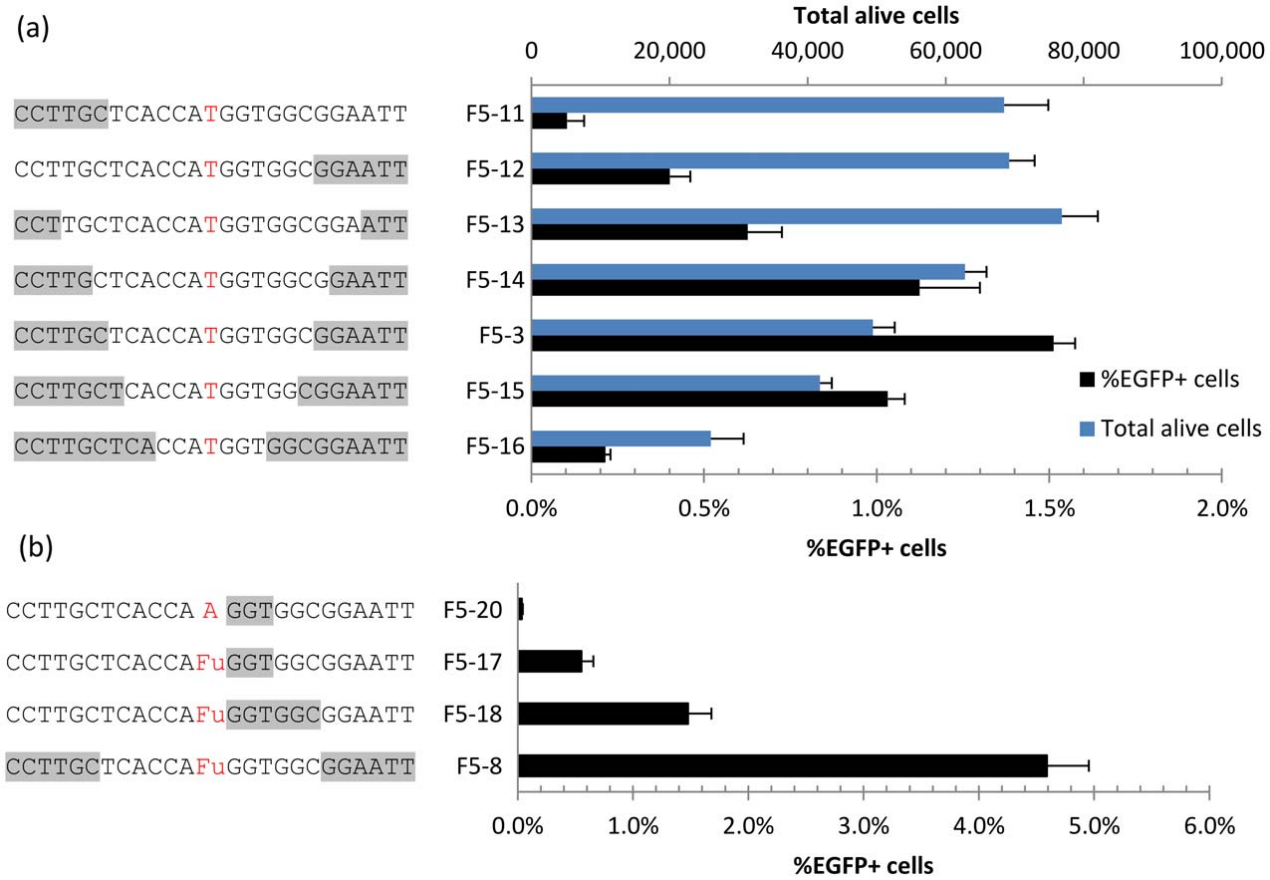
Varying PTOs in targeting oligos. All oligos in this figure are complementary to the non-transcribed strand’s first potential start codon TTG, similar to F5-3 in Fig. 1. (a) Oligo toxicity and targeting efficiency as a function of PTO modifications. PTO bond position shown in gray. ‘Total alive cells’ was estimated as the total gated PI- cells in 200 uL from a 24-well plate 48 hrs post oligo transfection. (b) Correction efficiencies of new oligo designs with reduced PTO bonds and using FU modified base. n = 4.

PTO modifications are used to protect oligos from nuclease degradation, however this modification has been reported to be toxic to cells [30]. To quantify this toxicity in relationship to *mEGFP* targeting efficiency we varied the number of PTO modifications in the targeting oligos and measured the effects on both cell survival and correction efficiency after 48 hrs. We found a clear trend where increased number of PTO bonds results in lower survival of cells 48 hrs after oligo transfection (Figure 3a), consistent with PTOs being toxic. In addition, an oligo with PTOs only at the 3′ end (F5-12) leads to higher correction efficiency than one with PTOs only at the 5′ end (F5-11, Fig. 3a). Interestingly, *mEGFP* correction steadily decreased with additional PTOs when there were more than six at each end. These results suggested that finding a balance between toxicity and efficiency might be necessary for generating stably corrected cells.

### Cells Corrected with Less-protected Oligos are More Likely to Proliferate

**Figure 4:**
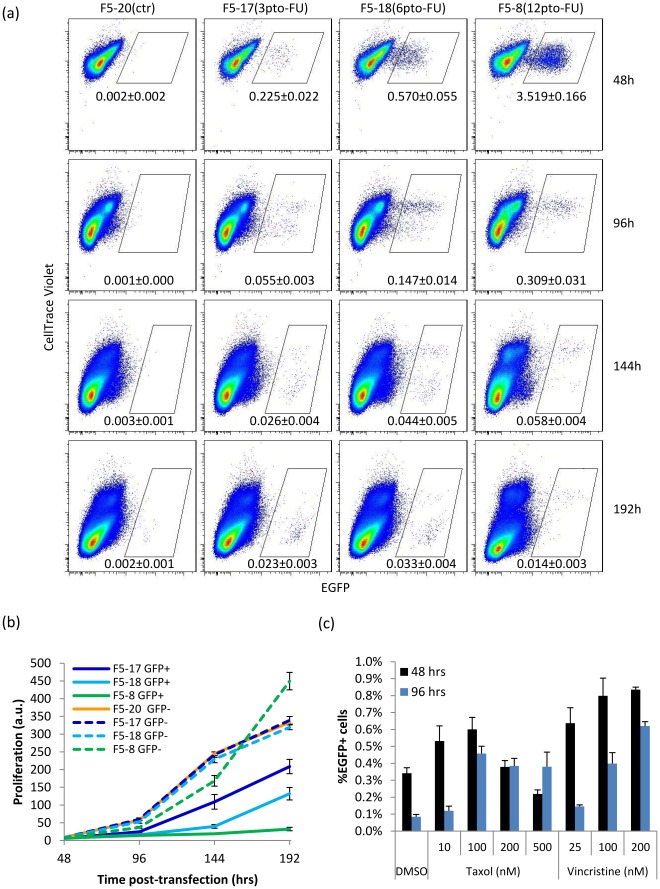
Proliferation defect in corrected cells. Proliferation is inversely proportional to the MFI of the CellTrace Violet dye. Cells were treated with CellTrace Violet dye, then split and transfected with oligos 48 hrs later. Cells were fixed at each time point and run together on a LSRfortessa cell analyzer at the end of the time course (a) Flow cytometry dot plots showing relative proliferation of EGFP+ and EGFP- cells generated with different targeting oligos, at four different time points post-transfection. %EGFP shown as average±s.d. n = 6. (b) Proliferation index is calculated as the inverse MFI of CellTrace ×10^6^ for each population. (c) Cells treated with anti-microtubule drugs Taxol and Vincristine 24 h after transfection with oligo F5-17 and 24 h exposure time, checked at 48 h and 96 h post-transfection. n = 4.

We hypothesized that reducing the number of PTO bonds might increase the number of proliferating EGFP+ cells. However, since reducing the number of PTO bonds also decreases targeting efficiency, we decided to concentrate on protecting the 3′ end. Furthermore, we decided to test internally protected oligos, since these have been found to lead to higher survival rates [20]. We designed two new oligos with three or six PTO bonds 3′ to the centrally located mismatch, all using a FU base analog (Table 1, Fig. 3b). Previous work has described the defect in oligo-corrected cells as a G2/M cell cycle arrest [20]–[21], [24], with few cells moving past it. In order to generate a more detailed view of the effects of oligos on proliferation, we tracked cells using the CellTrace Violet dye [31]–[32] (Figure 4a). This allowed us to follow the relative proliferation rate of the corrected (EGFP+) and uncorrected (EGFP-) cell populations by flow cytometry as the inverse dilution rate of the CellTrace dye’s Mean Fluorescent Intensity (MFI) (Figure 4b). First, we observe that in general EGFP+ cells proliferate slower than corresponding EGFP- cells. Second, the proliferation of EGFP+ cells inversely correlates with the number of PTO bonds of the oligo used to generate them, with the *EGFP*+ cells from the F5-8 (12 PTO) oligo hardly proliferating and the F5-18(6 PTO) having an intermediate phenotype. The proliferation rate of corrected cells depended on the number of PTO bonds on the oligo, suggesting it is the correcting oligo itself and not the expression of *EGFP* the cause of their proliferation defect. To functionally validate this observation, we treated F5-17 transfected cells with anti-microtubule drugs, which are preferentially toxic to proliferating cells [33], 24 hrs post-transfection (Fig. 4c). This produced an increase of EGFP+ cells at 48 and 96 hrs, suggesting that non-corrected cells had been preferentially inhibited, and therefore that corrected cells experience delayed proliferation. To verify that the effects of the anti-microtubule drugs were not due to endosome destabilization leading to increased oligo release, cell were treated with chloroquine, which actually slightly decreased targeting frequencies (not shown).

**Figure 5:**
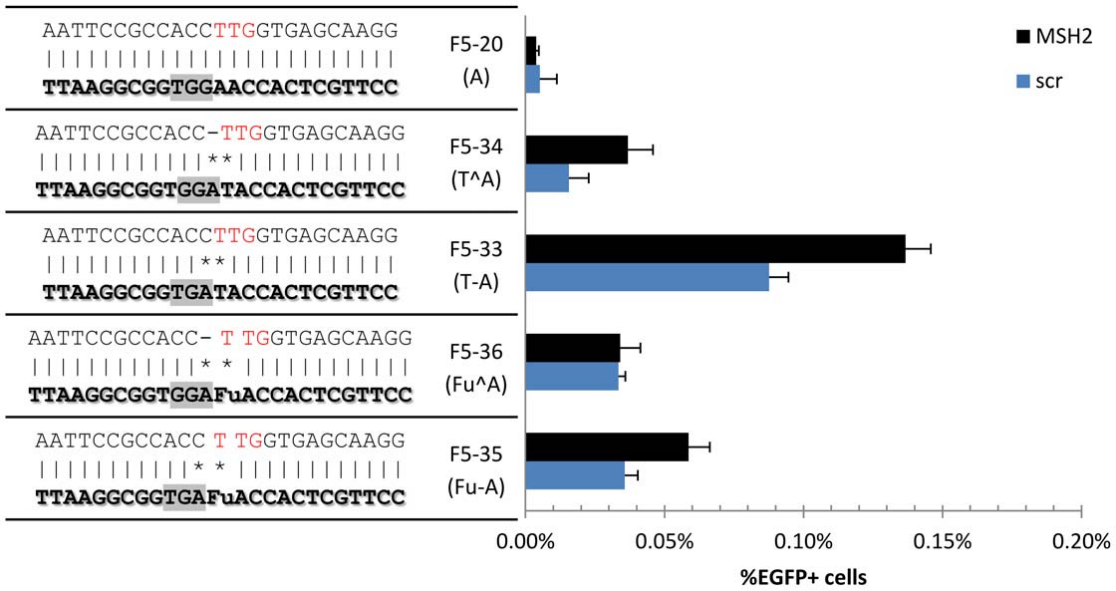
Generating double mutants. Oligos complementary to the first start codon in the non-transcribed strand and containing a second mismatch, either an insertion (∧A) or substitution (-A), with silenced MSH2 and assayed 36 hrs after transfection. Targeting oligos shown in duplex 3′-’to 5′-, with PTO highlighted in gray and mismatches shown with an asterisk. F5-20 was used as non-correcting control. n = 4.

In the case of the F5-17 oligo, the EGFP+ cells seemed to achieve a proliferation rate similar to the uncorrected *EGFP*- cells eight days post-transfection, since the percentage of positive cells remained constant between 144 and 192 hrs (Fig. 4a) and when checked again after a few weeks of passaging (not shown). By doubling the amount of lipofectamine:oligo complex we were able to double the frequency of corrected cells to ~0.05% eight days post-transfection (Figure S4). We chose this time point to repeat our single-cell sorting assay and found that EGFP+ cells generated with the F5-17 oligo easily generated clonal populations. These clones were verified by sequencing and found to have the desired genome modification (Figure S5a). To verify that this modification was not due just to spontaneous mutations, we repeated the experiment with oligos that introduced two adjacent nucleotide changes, one of which would restore the ATG start codon of *mEGFP* (using either a T or FU base) and other of which would introduce either a second substitution or a single-base insertion (Fig. 5). As expected, efficiency of incorporation of double-mutant oligos was significantly lower than for single-substitution oligos, with the double-substitution being more efficient than the single-substitution-and-insertion. Interestingly, using the FU base had an adverse effect in the double-substitution, suggesting a more complex mismatch recognition effect when more than one substitution is involved. Although the correction efficiencies were low, it was possible to sort EGFP+ cells generated with all four oligos, and all clones proliferated normally and were verified by sequencing to be correctly modified(Figure S5b,c).

**Figure 6:**
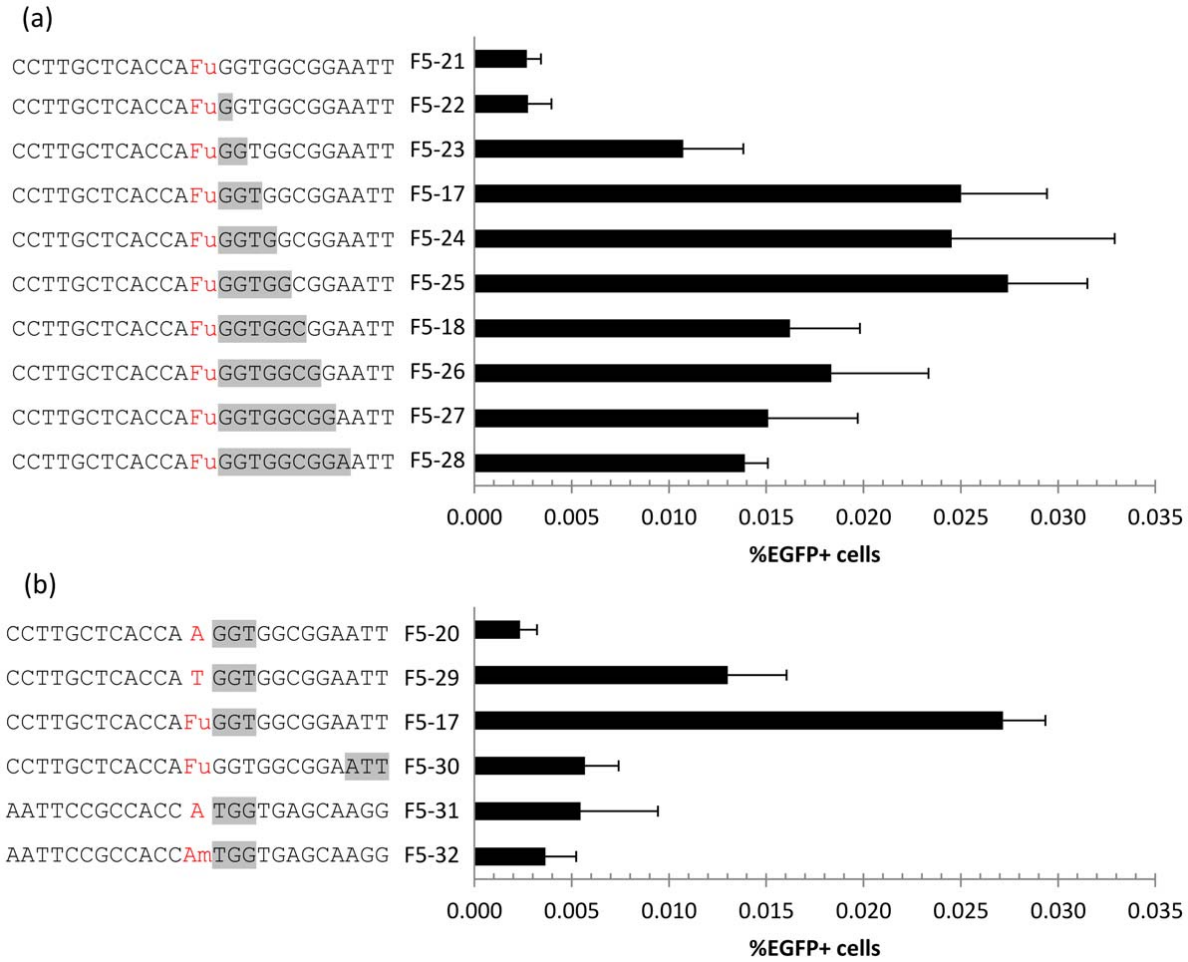
Effects of PTO in long-term survival of corrected cells. (a) Varying numbers of PTO bonds 3′ to the mismatch, shown in gray, suggest 3-5PTO bonds as optimal for balancing toxicity and stable targeting frequencies, assayed 14-days after oligo transfection by flow cytometry. (b) Testing the position of PTO bonds, strand targeted and use of modified bases in long term survival. F5-20 used as non correcting control, F5-17, -29 and -30 are complementary to the non-transcribed strand, while F5-31, -32 are complementary to the transcribed. n = 4.

To further elucidate oligo design principles, we checked for long-term survival of EGFP+ cells two weeks after transfection while varying the number of PTO bonds 3′ to the mismatch (Figure 6a). This showed 3-5PTO bonds as optimal, resulting in at least ~10-fold increase in targeting efficiency compared to unmodified oligos. We also varied the position of the PTO bonds, the strand polarity of the oligo used and the presence of modified bases (Figure 6b). When using an oligo complementary to the transcribed strand (F5-31, -32) we were able to detect some EGFP+ cells, but only slightly above the control oligo F5-20, suggesting the strand bias against the transcribed strand observed in this cell line is not just due to the additional DNA replication necessary to express the corrected gene when targeting this strand. Comparing the presence of 3PTO bonds at the 3′ terminus (F5-30) vs. 3′ internal to the mismatch (F5-17) produced a ~4-fold reduction in EGFP+ cells, confirming that internal PTO modifications result in higher stable targeting frequencies. Comparing the natural (F5-29) and modified (F5-17) base oligos resulted in a similar 2-fold increase for the modified base as when assayed 48 hrs after transfection. Thus, optimizing the oligo design with modified base analogs and reducing the number of PTO bonds enabled us to generate stable, isogenic populations genetically modified with oligos.

### The Proliferation Defect in Corrected Cells may be Associated to a Cellular Immune Response to Transfected Oligos

Our results suggest that the PTO modification in the oligos is the main reason corrected cells proliferate slower, rather than the mismatch generated by the oligo incorporation. Currently, the principal mechanism associated with PTO-modified oligo toxicity is the generation of DNA double-strand breaks. To check this we measured the levels of γ-H2AX phosphorylation in oligo transfected cells by flow cytometry (Figure S6), and find that reducing the number of PTO bonds reduced the levels of γ-H2AX phosphorylation. Interestingly, this also showed that EGFP+ cells had lower levels of γ-H2AX phosphorylation when compared with the non-corrected EGFP- cells treated with the same oligo. This suggests that although γ-H2AX phosphorylation may be a general marker of oligo toxicity, DNA double-strand breaks might not be the cause of decreased proliferation of oligo-modified EGFP+ cells, or at least not the only cause.

To further explore the proliferation defect in corrected cells, we sorted EGFP+ and EGFP- cells 36 hrs after transfection with the F5-17 oligo and performed RNA-seq to compare the two populations. We found a surprisingly high number of modestly differentially regulated genes (400 genes, p<10^−70^) between corrected and uncorrected cells (Dataset S1). We analyzed this top 400 gene list with ToppFun(http://toppgene.cchmc.org) to look for enrichment on any Gene Ontology categories. Interestingly ‘viral reproduction’ and ‘viral infectious cycle’ were among the most highly represented (Table S4).

**Figure 7:**
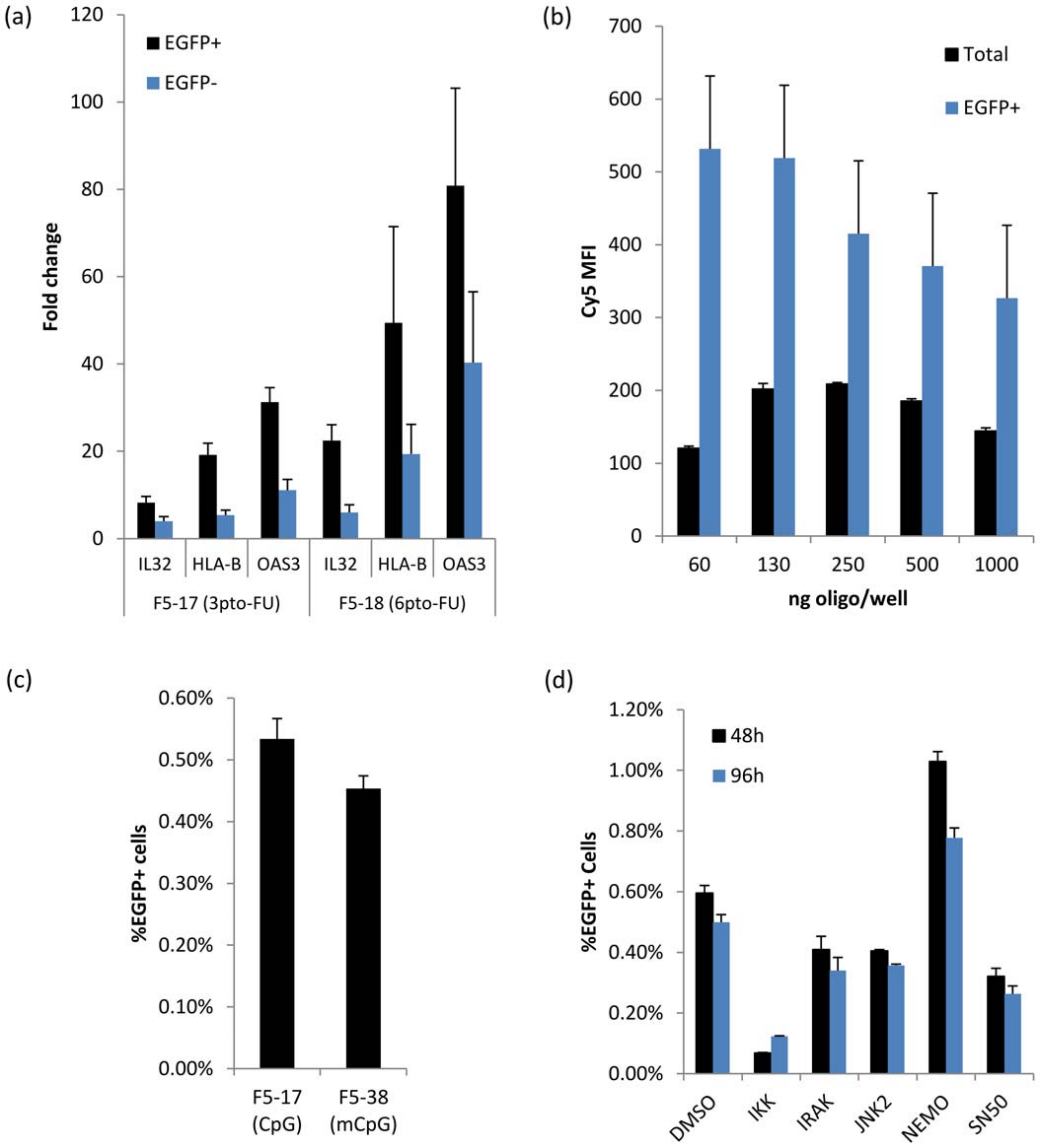
Induction of immune-related genes in oligo-transfected cells. (a)RT-qPCR relative quantification was performed for key immune-related genes, normalizing oligo-transfected to untransfected cells. (b) Mean Fluorescence Intensity (MFI) of total and EGFP+cells transfected with both F5-17 and F5-38 (Cy5-labeled) oligo at varying concentrations. Transfections were done in 24-well plates. n = 4 (c) Methylation of the CpG sequence present in the targeting oligo has no effect on the %EGFP+ cells. (d) Small-molecule inhibitors against key immune effectors, with DMSO as control, added 24 h after F5-17 oligo transfection assayed for EGFP+ cells at 48 h and 96 h. n = 4.

To further explore this observation, we choose three highly differentially expressed genes known to be involved in antiviral/cellular immune responses: *HLAB*, *IL32* and *OAS3*. We validated these genes by RT-qPCR, which allowed us to normalize the expression levels of corrected and uncorrected cells to untransfected cells(Fig. 7a). This revealed much higher induction levels for both populations relative to untransfected cells. For F5-17, the difference between transfected and untransfected cells was much higher (8–30 fold) than between EGFP+ and EGFP- cells (2–4 fold), suggesting that both non-corrected and corrected cells share an ‘oligo-transfected’ transcription profile. To verify that the gene changes were not due to EGFP itself, we checked the expression levels of EGFP+ and EGFP- cells after transfection with the F5-18 oligo. Like F5-17, F5-18 also generates EGFP+ cells, but F5-18 is a more toxic oligo as it contains three additional PTO bonds. If the upregulation of the three immune response genes we had observed were due to effects of the EGFP protein and not oligo toxicity, EGFP+ or EGFP- cells should have similar expression of these genes whether they had been transfected with F5-17 or F5-18. In contrast, however, the F5-18 transfected cells showed proportionally higher levels of induction of the three genes relative to F5-17, with EGFP+ cells still ~2–4 fold higher than EGFP- cells. One possible explanation for the difference between corrected and uncorrected cells would be that corrected cells got more oligos during transfection. We tested this by co-transfecting a targeting oligo along with a Cy5-labled one (F5-37). This showed that EGFP+ cells consistently had 2–5 times higher levels of oligos at varying oligo concentrations (Fig. 7b), suggesting that corrected cells may be proliferating less due to relatively higher oligo concentrations inducing a stronger immune response. Oligos with higher number of PTO modification have increased half-lives, which might lead to higher immune stimulation.

One possible cause for the immune response to the transfected oligos would be the presence of an unmethylated CpG in the oligo sequence. We tested an oligo with a methyl-C modification (F5-38, Table 1), but this had no effect on the percentage of EGFP+ cells after oligo transfection (Figure 7c). This is not unexpected however, since non-CpG-containing oligos have also been found to activate inflammatory responses [34]. Additionally, we tested small-molecule inhibitors targeting key immune signaling pathways, out of which NEMO inhibition produced a ~2-fold increase in the percentage of EGFP+ cells (Figure 7d). This increase, however, did not translate to higher survival eight days post-transfection(not shown). Further work is needed to determine the specific immune signaling pathway that might be induced in oligo-mediated targeting. Inhibiting such pathway may reduce the toxicity of PTO-modified oligos, leading to further improvements in targeting efficiencies.

## Discussion

In this work we describe our progress towards oligo-mediated genome engineering in human cells. An efficient version of this technology would be highly desirable since it does not require the design of custom proteins such as ZFNs or TALENs, simplifying experimental design and reducing time and costs. Currently, however, oligo-mediated genome engineering in human cells is severely limited by both low oligo incorporation efficiency and the low survival of modified cells. The nature of this limitation is likely multifactorial, with DNA damage being the most studied so far [21], [24]. Recent evidence in bacterial and mammalian systems suggests a model of oligo incorporation during DNA replication. Thus, when the oligo carrying the desired genetic change gets incorporated, it can be detected as a DNA replication error by the MMR machinery. The cause of low survival of corrected cells is unknown, but is at least partly caused by the cytotoxicity of the oligos.

To reduce the impact of MMR on oligo-mediated correction efficiency, we tested if using chemically modified base analogs capable of avoiding MMR recognition in *E. coli* would have a similar effect in human cells. As in *E. coli*, we found that FU and AM outperform thymine and adenine, respectively, in creating T-T or A-A mismatches leading to A/T transversions. By transiently silencing *MSH2* and *MLH1* we found that, at least for FU, the increased targeting efficiency of these modified bases is in part due to avoidance of the MMR machinery. This effect, however, seems to be complex since adding a second mismatch produced variable results (Figure 5), likely due to sequence context effects [35]. Because our reporter assay is limited to restoring the start codon of *mEGFP*, we were only able to study modified bases substituting A and T, but we expect that other modified bases found to work in *E. coli* will have a similar effect. Further work is required to fully elucidate the mechanism by which modified bases increase targeting frequencies in different human cell types. This is especially true for the modified base FA, since it is being recognized more strongly by MMR than the native A base, yet it is still capable of increasing targeting efficiencies. It is possible that other MMR components, or additional DNA repair pathways such as NER might be responsible for recognizing this base, as it has been suggested that these two pathways may functionally overlap [36]–[37]. An advantage of using modified bases rather than suppressing the endogenous MMR machinery to increase oligo incorporation efficiency is the avoidance of unwanted mutations elsewhere in the genome that MMR suppression is likely to lead to [38], essential for functional genomics and potential gene therapy applications.

Using FU for MMR-avoidance in an oligo containing 6PTO bonds on each end lead to a substantial number (~3–5%) of EGFP+ cells shortly after transfection, however this EGFP+ population did not proliferate further. This suggests that a process other than MMR-mediated DNA damage signaling may be causing the low survival of corrected cells. Since PTO bonds have been previously implicated in oligo cytotoxicity, we tested varying the number and position of PTO bonds. We found a clear trend where a higher number of PTO bonds lowers the total number of cells surviving after transfection. Additionally, protecting only the 3′ end yielded a higher percentage of corrected cells than a similarly protected and similarly toxic 5′ end. Based on this, we designed two new oligos with a reduced number of PTO bonds 3′ to the mismatch and used these to examine cell proliferation after transfection in more detail. Using a cell tracking dye we found that the proliferation of corrected cells was consistently lower than that of uncorrected ones, and that the proliferation of corrected cells was increased when the targeting oligo contained a lower number of PTO bonds. The F5-17 oligo allowed us to isolate stably modified EGFP+ cells with the correct genotype. This oligo’s three PTO bonds are situated immediately 3′ to the mismatch base and are therefore internal to the oligo. The oligo would be reduced to only 16 bases long by 3′-to-5′ exonucleases, which might make annealing inefficient. We speculate instead that internal-3′ protection might prevent removal of oligos by proofreading polymerases after annealing while still allowing sufficient clearance of excess oligo by autonomous exo- and endonucleases. Supporting this, F5-30, a similar oligo but with the 3 PTO bonds at the 3′ terminal resulted in a ~4-fold decrease in survival compared to F5-17. To try and elucidate the cause of oligo toxicity in relation to PTO bond number we measured the levels of γ-H2AX phosphorylation. We find that the number of PTO bonds directly correlates to the levels of γ-H2AX phosphorylation, but EGFP+ cells have consistently lower levels than EGFP- cells, which counters observations on other cell lines [24]–[25]. The fact that HeLa cells have abnormal p53 signaling might account for the observation that EGFP+ cells have lower levels of γ-H2AX phosphorylation if, for example, a significant proportion of the observed double-strand breaks were an indirect consequence of oligos inducing apoptosis [39] rather than the breaks being directly caused by the oligos. Future investigation into the mechanisms of PTO toxicity and targeting oligo protection would be desirable.

The dot plots from our cell proliferation experiments show that modified cells are a subset of the population with a proliferation defect. To get a better understanding of this growth phenotype we did RNA-seq to compare the transcription profiles between corrected and uncorrected cells. This revealed a large number of mildly differentially expressed genes between the two populations, especially genes involved in immune/viral processes, based on GO category enrichment. Interestingly, DNA damage-associated GO categories had a much less significant p-value (~10^−2^ vs. ~10^−30^), further arguing against DNA damage causing the low survival of corrected cells. We validated the differential expression of *HLAB*, *OAS3* and *IL32* to use as immune response markers by RT-qPCR, which in addition allowed us to compare expression levels to those of untransfected cells and of cells treated with different targeting oligo designs. This uncovered two important clues: first, both corrected and uncorrected cells showed a much higher fold induction of immune genes than untransfected cells and second, more oligo PTO modifications lead to proportionally higher expression levels. Since cells that get more oligos during transfection are presumably more likely to become EGFP+, we believe that higher oligo concentrations within corrected cells may be inducing a stronger cellular immune response. A similar effect may also explain the toxicity of PTO modifications, since PTO modifications stabilize oligos and will therefore lead to higher sustained oligo concentrations after transfection, which in turn may lead to higher immune signaling. Alternatively, it is possible that cellular DNA sensors might recognize the abnormal PTO backbone itself [40]. Functional studies are currently underway to further explore this observation. The model of oligo-mediated immune response seems plausible, especially in light of the growing list of cellular immune DNA sensors [40]–[42] and the role of 3′ exonucleases in inflammatory diseases [43]. It is possible that the immune response triggered in oligo corrected cells might affect cellular phenotype in such a way that it confounds the effects of the targeted mutation itself. This could be assayed by expanding our comparison of transcriptional profiles of modified clonal populations to include comparisons against profiles of the unmodified ‘parental’ line. If necessary and depending on the intended study, mutations of interest could be evaluated in parallel with silent mutations to control for the modification process. Further work is necessary to better understand the roles of DNA double-strand breaks and cellular immunity in oligo-mediated toxicity, including testing these observations in different cell lines.

The wealth of sequencing projects and GWAS’s data will necessitate novel methods for experimentally testing human genetic variants. For modeling a few high-interest single-loci isogenic changes by targeted manipulation of human cell genomes, the current state-of the-art method of ZFNs + ssOligo [8] performs well. However, screening of hundreds of polymorphisms and their potential combinatorial interactions for phenotypic effects is not currently feasible with these methods. An oligo-mediated genome engineering method as used in *E. coli* [16] is far better suited for this, where up to ten simultaneous oligo incorporations are possible (Carr P.A. and Wang H.H., in revision), and experimental design is trivial and without the need of custom DNA-binding proteins. We estimate our current targeting frequency to be ~0.05% for HeLa cells. This level of targeting efficiency should easily allow the generation of isogenic cells by using a pooled genotyping serial enrichment strategy [29] [38]. The cellular immune response to transfected oligos described here represents a novel and exciting avenue which might lead to further reduction of toxicity and thus improved targeting efficiencies in multiple cell types. We are working to develop oligo-mediated genome engineering in human induced pluripotent stem cells, which could enable combinatorial testing of genetic variants in multiple tissues simultaneously, greatly expediting the study of human genetic variation and the genetic basis of human disease.

## Materials and Methods

### Cell Culture and Transfections

The HeLa F5 cell line was kindly provided by Dr. Depei Liu. These were cultured in DMEM with GlutaMAX and HEPES, supplemented with 10% HI-FBS, 100 mg/ml streptomycin and 100 U/ml penicillin (Invitrogen) at 37°C and 5% CO_2_ in a humidified incubator. Cells densities were determined using the Countess Automated Cell Counter (Invitrogen). For subculturing, cells were washed with PBS pH 7.4, trypsinized with TrypLE Express(Invitrogen) and neutralized with 10% HI-FBS DMEM.

### Oligo Transfections

HPLC-purified oligos were ordered from Integrated DNA Technologies, resuspended in distilled water and their concentrations verified by NanoDrop (Thermo Scientific). Cells were pre-seeded at 3×10^6^ cells per 10 cm plate two days before transfection. Pre-seeded cells were split to 100,000 cells/well for a 24-well plate or at 3×10^6^ cells for a 10 cm plate. The next day, cells were washed with PBS and 10% HI-FBS with no antibiotics was added. Transfections were done with Lipofectamine 2000 (Invitrogen) using a 3∶1 ratio of µL lipofectamine to µg oligo. For 24-well plates, 0.5 µg of oligo and 1.5 µL lipofectamine were diluted in 25 µL OptiMEM(Invitrogen) each, incubated at room temperature for 5 mins, combined by pipetting, then incubated again for 20 mins. The oligo complexes were added to cells dropwise and cultured continuously for 36–48 hrs. Unless otherwise noted, 20 µM thymidine was added to the cells two hours prior transfection.

### Flow Cytometry Analysis

After 36–48 hrs, oligo-transfected cells were washed with PBS, trypsinized, neutralized with 10% HI-FBS DMEM and transferred to 96-well U-bottom plate. Death exclusion was done with 10% propidium iodide (Roche), and total surviving cells were estimated as the total number of living cells counted at a determined volume. The plate was run on a five-laser BD LSRFortessa HTS with FACS Diva 6.1 software. EGFP was detected with the 488 nm laser, 530/30 nm filter; CellTrace Violet with the 405 nm laser, 450/50 nm filter; propidium iodide with the 561 nm, 610/20 nm filter; Cy5 with 640 nm laser, 670/30 nm filter. Cell sorting was performed on a BD FACSAria II SORP equipped with 375 nm, 405 nm, 445 nm, 488 nm, 561 nm, and 633 nm lasers.

### Genotyping and Sequencing

We used allele-specific realtime PCR to quantify corrected versus uncorrected *mEGFP* DNA in cells after oligo targeting. A 152 bp amplicon was amplified using a forward primer whose 3′ terminal base matched either the uncorrected or corrected *mEGFP* target site. Using standard curves for each primer pair, a ratio of corrected to non-corrected template strands could be estimated for each cell population. EGFP+ and EGFP- cells were sorted by FACS and pelleted. To lyse the cells, ~1,500 cells resuspended in 8.9 µl water were added to 1 µl 10× gold buffer and 0.1 µL prepGEM enzyme (ZyGEM) and incubated for 75°C for 5 min then 95°C for 5 min. The reaction was split into 2×5 µL and each aliquot was mixed with 20 µL PCR mix containing the corrected-specific or non-corrected-specific primer pair. PCR mix: 18.8 µL 1.1X Platinum Taq Supermix (Invitrogen), 0.5 µL each primer (stock solution 10 uM), 0.2 µL SYBR green I (stock solution a 1∶500 dilution of original tube). Realtime PCR was carried out on an Opticon 2 DNA Engine (MJ Research), under the following cycling conditions: 95°C for 3 min followed by 40 cycles of 95°C 30 sec, 62°C 30 sec, 72°C 30 sec. To sequence the *mEGFP* target site in stable non-corrected and corrected cell clones, a similar PCR protocol was used except with primers N1 and N3 from [27] that span the targeted region. Single-cells were sorted eight days post-oligo transfection, and clonal populations were obtained after growing for ~2 weeks. PCR products were sequenced by Genewiz, inc. All PCR primer sequences used can be found in the Table S1.

### RNA Interference

HeLa F5 cells were seeded 3×10^6^ cells per 10 cm plate. The next day, media was changed to 4 mL 10% HI-FBS with no antibiotic. Transfection of validated shRNA plasmids (Sigma-Aldrich) or scramble control (Addgene) was done with FuGENE HD, diluting 15 µg of HiSpeed Maxi-Prep(QIAGEN) purified plasmid and 60 µL of FuGENE HD(Roche) in 500 µL OptiMEM each. The dilutions were mixed by pipetting and incubated for 15 mins before adding complexes dropwise to plates. After overnight transfection, cells were washed, trypsinized and re-seeded in 10 cm plates with 10% HI-FBS DMEM and 3 µg/µL puromycin (Sigma-Aldrich) After two days of puromycin selection, cells were split into 24-well plates 100,000 cells/well for oligo transfections. shRNA target sequences can be found in Table S2.

### Cell Proliferation Assay

HeLa F5 cells seeded at 3×10^6^ cell per 10 cm plate were washed and incubated in 5 mL PBS with 10 µM CellTrace Violet(Invitrogen) for 10 mins at 37°C, washed and grown in 10% HI-FBS DMEM overnight. Cells were split and pre-seeded at 3×10^6^ cell per 10 cm plate, then the next day seeded in 24-well plates for oligo transfection. At each timepoint, cells were trypsinzed, centrifuged at 500 g for 5 mins and fixed in 4% PFA (Biolegend) for 20 min in the dark at room temperature. After another centrifugation, cells were resuspended in Cell Staining Buffer (Biolegend) and kept at 4°C. After the last timepoint samples were run together on a BD LSRFortessa HTS.

### Anti-microtubule Drug Treatment

Twenty-four hours after cells were transfected with oligos, media was changed and cells were exposed to varying concentrations of either Taxol or Vincristine (EMD) for 24 hours, then washed and assayed with the BD LSRFortessa HTS immediately or two days later (96 hours after oligo transfection).

### RNA-seq and qPCR

HeLa F5 cells plated at 3×10^6^ cells on a 10 cm plate were washed and 4 mL of OptiMEM with 20 µM thymidine was added two hours prior transfection with 15 µg oligo/45 µL lipofectamine. Thirty-six hours after transfection cells were trypsinized, filtered through a 35 µm cell strainer (BD Bioscience #352235), counted and centrifuged at 250 g for 5 mins. Cells were resuspended at a density of 1×10^7^ cells/mL in 20% HI-FBS DMEM and sorted into EGFP+ and EGFP- directly into Trizol LS(Invitrogen). Extracted RNA was precipitated and concentrated on a RNAeasy Micro kit column (QIAGEN ), and the quality of RNA was determined in an Agilent 2100 Bioanalyzer. Each sorting was done from 15 10-cm plates of transfected cells and the RNA of three independent sorting sessions was pooled to ~2 µg of total RNA. RNA-seq was performed as described [44] with some modifications, i.e. there was no normalization of the library. Reads were aligned using TOPHAT and reads mapping to each gene were analyzed for differential expression with a Bayesian statistic [45]. For RT-qPCR, first-strand synthesis was done with the SuperScript III kit (Invitrogen), and qPCR with the KAPA SYBR Fast Universal 2× mix following manufacturer’s protocol. The sequence of the IDT-synthesized qPCR oligos can be found in Table S2. The reaction was assayed in a DNA Engine Opticon2 instrument (MJ Research).

### Small Molecule Immune Inhibitors Drug Treatment

After cells were transfected with oligos for 24 hours, media was changed and cells were exposed 5 µM IKK inhibitor VI, 50 µM IRAK1/4 inhibitor, 20 µM Cell-Permeable NEMO-Binding Domain Binding Peptide, NF-κB SN50, 36 µM Cell-Permeable Inhibitor Peptide and 10 µM JNK Inhibitor II (EMD) for 24 hours then assayed with the BD LSRFortessa HTS immediately or two days later (96 hours after oligo transfection).

## Supporting Information

Figure S1 **Nuclear localization of transfected oligos.** Cells were grown in glass-bottom 24-well plates and stained with Hoechst 33342 10 ug/mL. The next day, cells were transfected with 500 ng fluorescein-labeled oligo using various cationic lipid reagents following manufacturer instructions and imaged 24 hours after transfection. Scale bar = 10 µm ea.
(TIFF)

Figure S2 **Oligo delivery and correction efficiencies using various transfection reagents.** Cells were plated 100,000 cells/well on a 24-well plate, n = 4. (a) Cells were transfected with a fluorescein-labeled oligo, and oligo delivered is shown as the MFI of the total cell population. (b) Correction frequencies with oligo F5-8 as a function of oligo amount.
(TIFF)

Figure S3 **RNAi validation.** (a) Western Blotting confirming knockdown of MMR components. Primary antibodies anti-MSH2 (ab52266, 1∶5,000), anti-MLH1 (ab92312, 1∶2,000) and anti-Actin (ab3280, 1∶10,000), and secondary antibodies anti-rabbit IgG (ab6721, 1∶25,000) and anti-mouse IgG (ab6728 1:25,000) were obtained from Abcam. (b), (c) siRNA targeting mismatch repair components have a lower effect with modified bases. A 24-well plate was treated with 80 nM siRNA, 1 uL RNAiMax following manufacturer’s protocol. siRNA-treated cells were transfected with targeting oligos 72 hrs later. n = 4 siRNAs sequences can be found in Table S3.
(TIFF)

Figure S4 **HeLa F5 cells transfected with F5-17 oligo, either 1x or 2x DNA:lipofectamine complexes, assayed for %EGFP+ cells eight days after transfection.**
(TIFF)

Figure S5 **Sequencing corrected EGFP+ clonal cells.** Single-cells were sorted into a 96-well plate 8 days post-transfection with (a) F5-17,(b)F5-34, (c)F5-35, grown for two weeks and then sequenced. Sequence spectrum overlap shows one of the two mEGFP copies was modified.
(TIFF)

Figure S6 **γ-H2AX phosphorylation staining.** Cells were plated 500,000/well on a 6-well plate, then transfected with the corresponding oligos. After 36 hrs, cells were washed, fixed in 4%PFA, permeabilized with 10% saponin, then stained with Alexa Fluor 647 anti-H2A.X-Phosphorylated Antibody (1∶25, Biolegend) shaking for 20 mins at room temperature.
(TIF)

Table S1 **PCR primers sequences.**
(DOCX)

Table S2 **shRNAs sequences.**
(DOCX)

Table S3 **siRNA sequences.**
(DOCX)

Table S4 **Enriched GO categories.** Top 400 genes based on p-value were analyzed with Toppgene, showing strong representation of viral process category.
(DOCX)

Dataset S1 **Top 400 hits from RNA-seq experiment.** GFP+ and GFP- refers to cells after treating with oligo F5-17.
(XLSX)

## References

[1] Mardis ER (2006). Anticipating the $1,000 genome.. Genome Biology.

[2] von Bubnoff A (2008). Next-Generation Sequencing: The Race Is On.. Cell.

[3] Freedman ML, Monteiro ANA, Gayther SA, Coetzee GA, Risch A (2011). Principles for the post-GWAS functional characterization of cancer risk loci.. Nat Genet.

[4] Vasquez KM, Marburger K, Intody Z, Wilson JH (2001). Manipulating the mammalian genome by homologous recombination.. Proceedings of the National Academy of Sciences.

[5] Pickrell JK, Marioni JC, Pai AA, Degner JF, Engelhardt BE (2010). Understanding mechanisms underlying human gene expression variation with RNA sequencing.. Nature.

[6] Urnov FD, Miller JC, Lee Y-L, Beausejour CM, Rock JM (2005). Highly efficient endogenous human gene correction using designed zinc-finger nucleases.. Nature.

[7] Hockemeyer D, Wang H, Kiani S, Lai CS, Gao Q (2011). Genetic engineering of human pluripotent cells using TALE nucleases.. Nat Biotechnol.

[8] Soldner F, Laganière J, Cheng AW, Hockemeyer D, Gao Q (2011). Generation of Isogenic Pluripotent Stem Cells Differing Exclusively at Two Early Onset Parkinson Point Mutations.. Cell.

[9] Maeder ML, Thibodeau-Beganny S, Sander JD, Voytas DF, Joung JK (2009). Oligomerized pool engineering (OPEN): an “open-source” protocol for making customized zinc-finger arrays.. Nat Protocols.

[10] Chandrasekharan S, Kumar S, Valley CM, Rai A (2009). Proprietary science, open science and the role of patent disclosure: the case of zinc-finger proteins.. Nature biotechnology.

[11] Move over ZFNs (2011). Nat Biotech. http://dx.doi.org/10.1038/nbt.1935.

[12] Şöllü C, Pars K, Cornu TI, Thibodeau-Beganny S, Maeder ML (2010). http://nar.oxfordjournals.org/content/early/2010/08/13/nar.gkq720.

[13] Doyon Y, Vo TD, Mendel MC, Greenberg SG, Wang J (2010). Enhancing zinc-finger-nuclease activity with improved obligate heterodimeric architectures.. Nature Methods.

[14] Carr PA, Church GM (2009). Genome engineering.. Nature biotechnology.

[15] Ellis HM, Yu D, DiTizio T (2001). High efficiency mutagenesis, repair, and engineering of chromosomal DNA using single-stranded oligonucleotides.. Proceedings of the National Academy of Sciences.

[16] Wang HH, Isaacs FJ, Carr PA, Sun ZZ, Xu G (2009). Programming cells by multiplex genome engineering and accelerated evolution.. Nature.

[17] Aarts M, te Riele H (2010). Progress and prospects: oligonucleotide-directed gene modification in mouse embryonic stem cells: a route to therapeutic application.. Gene Ther.

[18] Olsen PA, Randøl M, Luna L, Brown T, Krauss S (2005). Genomic sequence correction by single-stranded DNA oligonucleotides: role of DNA synthesis and chemical modifications of the oligonucleotide ends.. The Journal of Gene Medicine.

[19] Wu XS, Xin L, Yin WX, Shang XY, Lu L (2005). Increased efficiency of oligonucleotide-mediated gene repair through slowing replication fork progression.. Proceedings of the National Academy of Sciences.

[20] Papaioannou I, Disterer P, Owen JS (2009). Use of internally nuclease-protected single-strand DNA oligonucleotides and silencing of the mismatch repair protein, MSH2, enhances the replication of corrected cells following gene editing.. J Gene Med.

[21] Olsen PA, Randol M, Krauss S (2005). Implications of cell cycle progression on functional sequence correction by short single-stranded DNA oligonucleotides.. Gene Ther.

[22] Radecke S, Radecke F, Peter I, Schwarz K (2006). Physical incorporation of a single-stranded oligodeoxynucleotide during targeted repair of a human chromosomal locus.. The Journal of Gene Medicine.

[23] Dekker M, Brouwers C, te Riele H (2003). Targeted gene modification in mismatch-repair-deficient embryonic stem cells by single-stranded DNA oligonucleotides.. Nucleic Acids Research.

[24] Olsen PA, Solhaug A, Booth JA, Gelazauskaite M, Krauss S (2009). Cellular responses to targeted genomic sequence modification using single-stranded oligonucleotides and zinc-finger nucleases.. DNA repair.

[25] Aarts M, te Riele H (2010). Subtle gene modification in mouse ES cells: evidence for incorporation of unmodified oligonucleotides without induction of DNA damage.. http://dx.doi.org/10.1093/nar/gkq589.

[26] Wang HH, Xu G, Vonner AJ, Church G (2011). http://nar.oxfordjournals.org/content/early/2011/05/23/nar.gkr183.

[27] Yin WX, Wu XS, Liu G, Li ZH, Watt RM (2005). Targeted correction of a chromosomal point mutation by modified single-stranded oligonucleotides in a GFP recovery system.. Biochemical and Biophysical Research Communications.

[28] Disterer P, Simons JP, Owen JS (2009). Validation of oligonucleotide-mediated gene editing.. Gene Therapy.

[29] Aarts M, Dekker M, Dekker R, de Vries S, van der Wal A (2009). Gene modification in embryonic stem cells by single-stranded DNA oligonucleotides.. Methods in molecular biology (Clifton, NJ).

[30] Liu C, Wang Z, Huen MSY, Lu L-Y, Liu D-P (2009). Cell Death Caused by Single-Stranded Oligodeoxynucleotide-Mediated Targeted Genomic Sequence Modification.. Oligonucleotides.

[31] Efimova E, Martinez O, Lokshin A, Arima T, Prabhakar BS (2003). IG20, a MADD Splice Variant, Increases Cell Susceptibility to γ-Irradiation and Induces Soluble Mediators That Suppress Tumor Cell Growth.. Cancer Research.

[32] Hawkins ED, Hommel M, Turner ML, Battye FL, Markham JF (2007). Measuring lymphocyte proliferation, survival and differentiation using CFSE time-series data.. Nat Protocols.

[33] Engstrom JU, Suzuki T, Kmiec EB (2009). Regulation of targeted gene repair by intrinsic cellular processes.. BioEssays.

[34] Senn JJ, Burel S, Henry SP (2005). Non-CpG-Containing Antisense 2′-Methoxyethyl Oligonucleotides Activate a Proinflammatory Response Independent of Toll-Like Receptor 9 or Myeloid Differentiation Factor 88.. Journal of Pharmacology and Experimental Therapeutics.

[35] Mazurek A, Johnson CN, Germann MW, Fishel R (2009). Sequence context effect for hMSH2-hMSH6 mismatch-dependent activation.. Proceedings of the National Academy of Sciences.

[36] Fleck O, Lehmann E, Sch|[auml]|r P, Kohli J (1999). Involvement of nucleotide-excision repair in msh2 pms1-independent mismatch repair.. Nature Genetics.

[37] Huang JC, Hsu DS, Kazantsev A, Sancar A (1994). Substrate spectrum of human excinuclease: repair of abasic sites, methylated bases, mismatches, and bulky adducts.. Proceedings of the National Academy of Sciences.

[38] Dekker M, de Vries S, Aarts M, Dekker R, Brouwers C (2011). Transient suppression of MLH1 allows effective single-nucleotide substitution by single-stranded DNA oligonucleotides.. Mutation Research/Fundamental and Molecular Mechanisms of Mutagenesis.

[39] Rogakou EP, Nieves-Neira W, Boon C, Pommier Y, Bonner WM (2000). Initiation of DNA Fragmentation during Apoptosis Induces Phosphorylation of H2AX Histone at Serine 139.. Journal of Biological Chemistry.

[40] Burckstummer T, Baumann C, Bluml S, Dixit E, Durnberger G (2009). An orthogonal proteomic-genomic screen identifies AIM2 as a cytoplasmic DNA sensor for the inflammasome.. Nat Immunol.

[41] Zhang X, Brann TW, Zhou M, Yang J, Oguariri RM (2011). Cutting Edge: Ku70 Is a Novel Cytosolic DNA Sensor That Induces Type III Rather Than Type I IFN.. The Journal of Immunology.

[42] Barber GN (2011). Innate immune DNA sensing pathways: STING, AIMII and the regulation of interferon production and inflammatory responses.. Current Opinion in Immunology.

[43] Coscoy L, Raulet DH (2007). DNA Mismanagement Leads to Immune System Oversight.. Cell.

[44] Christodoulou DC, Gorham JM, Herman DS, Seidman JG (2011). Construction of Normalized RNA-seq Libraries for Next-Generation Sequencing Using the Crab Duplex-Specific Nuclease.. Curr Protoc Mol Biol.

[45] Audic S, Claverie J-M (1997). The Significance of Digital Gene Expression Profiles.. Genome Research.

