# Supplementary figures and images for: Stable Gene Targeting in Human Cells Using Single-Strand Oligonucleotides with Modified Bases
Source: PLoS One. 2012 May 14;7(5):e36697. doi: 10.1371/journal.pone.0036697 (PMC3351460; doi:10.1371/journal.pone.0036697)

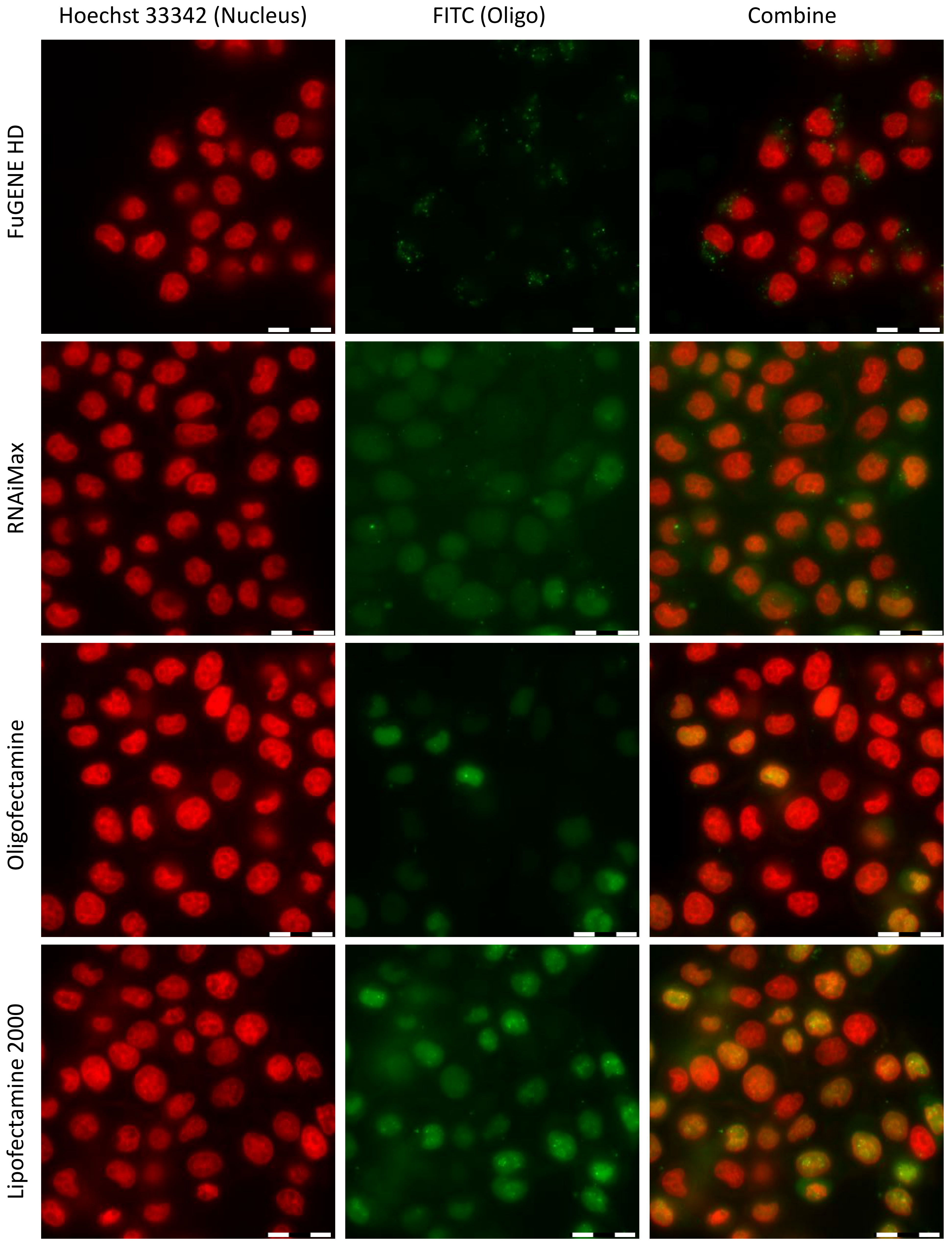

Supplement: Figure S1 — Nuclear localization of transfected oligos. Cells were grown in glass-bottom 24-well plates and stained with Hoechst 33342 10 ug/mL. The next day, cells were transfected with 500 ng fluorescein-labeled oligo using various cationic lipid reagents following manufacturer instructions and imaged 24 hours after transfection. Scale bar = 10 µm ea. (TIFF) [file pone.0036697.s001.tif]

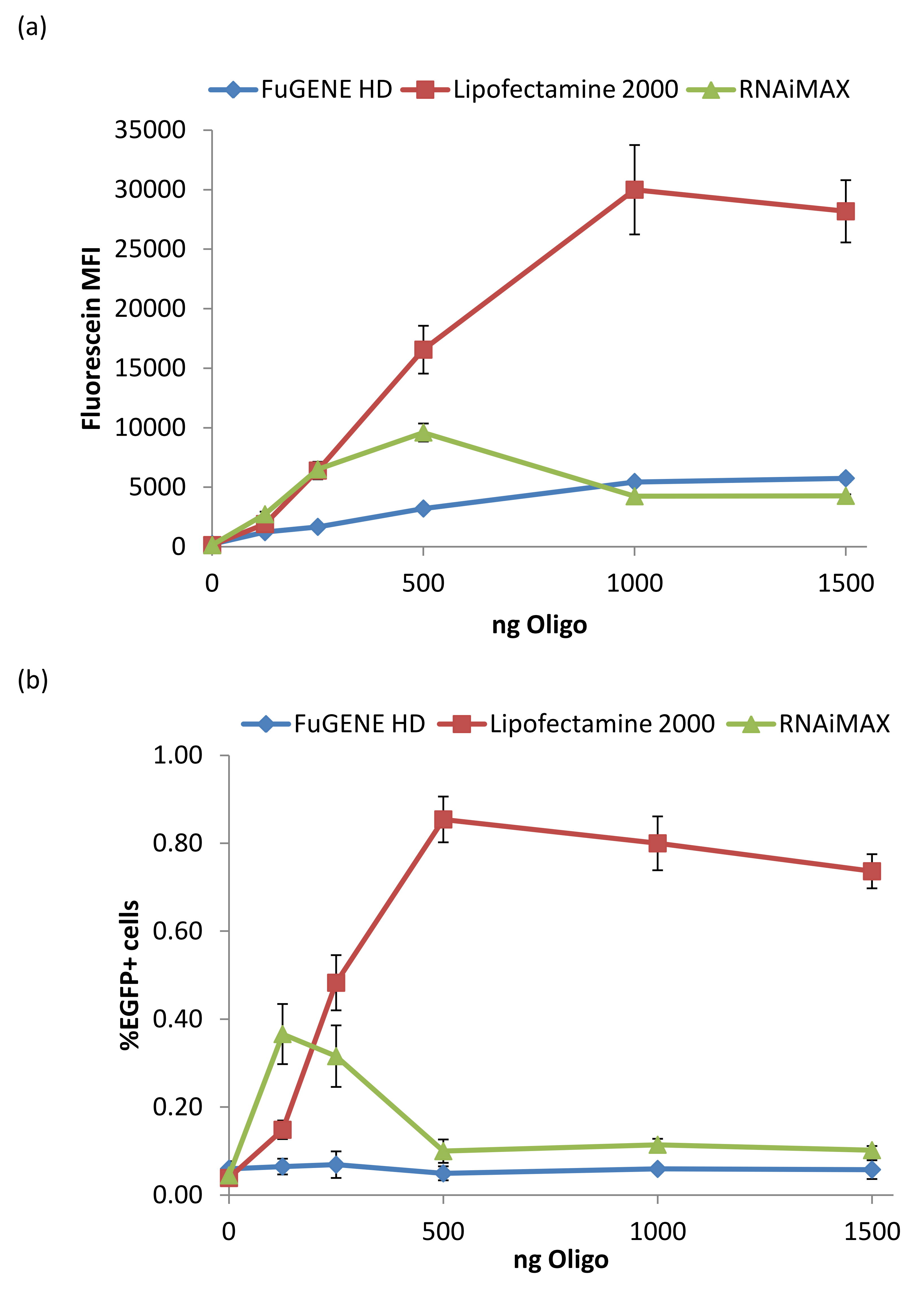

Supplement: Figure S2 — Oligo delivery and correction efficiencies using various transfection reagents. Cells were plated 100,000 cells/well on a 24-well plate, n = 4. (a) Cells were transfected with a fluorescein-labeled oligo, and oligo delivered is shown as the MFI of the total cell population. (b) Correction frequencies with oligo F5-8 as a function of oligo amount. (TIFF) [file pone.0036697.s002.tif]

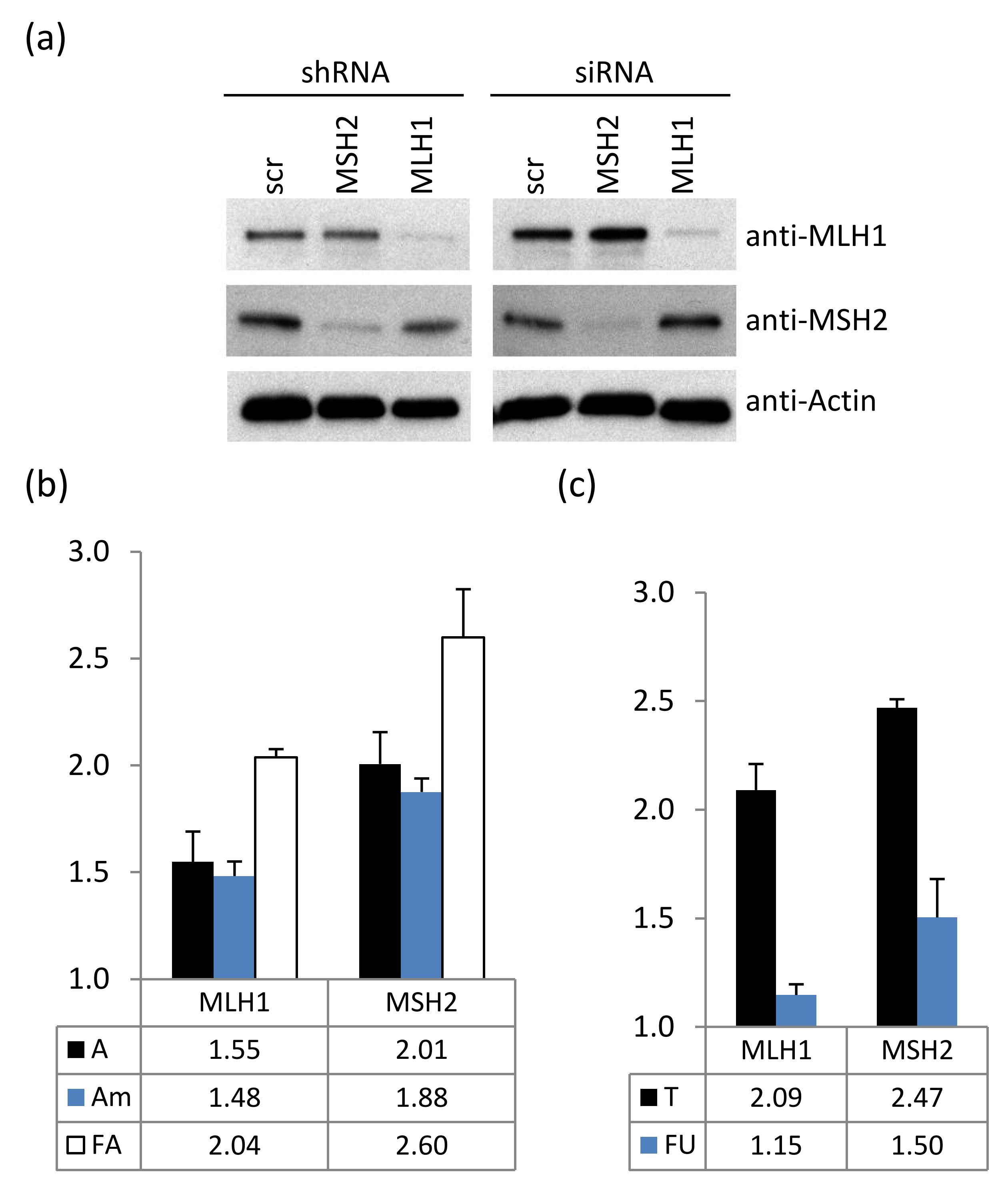

Supplement: Figure S3 — RNAi validation. (a) Western Blotting confirming knockdown of MMR components. Primary antibodies anti-MSH2 (ab52266, 1∶5,000), anti-MLH1 (ab92312, 1∶2,000) and anti-Actin (ab3280, 1∶10,000), and secondary antibodies anti-rabbit IgG (ab6721, 1∶25,000) and anti-mouse IgG (ab6728 1:25,000) were obtained from Abcam. (b), (c) siRNA targeting mismatch repair components have a lower effect with modified bases. A 24-well plate was treated with 80 nM siRNA, 1 uL RNAiMax following manufacturer’s protocol. siRNA-treated cells were transfected with targeting oligos 72 hrs later. n = 4 siRNAs sequences can be found in Table S3. (TIFF) [file pone.0036697.s003.tif]

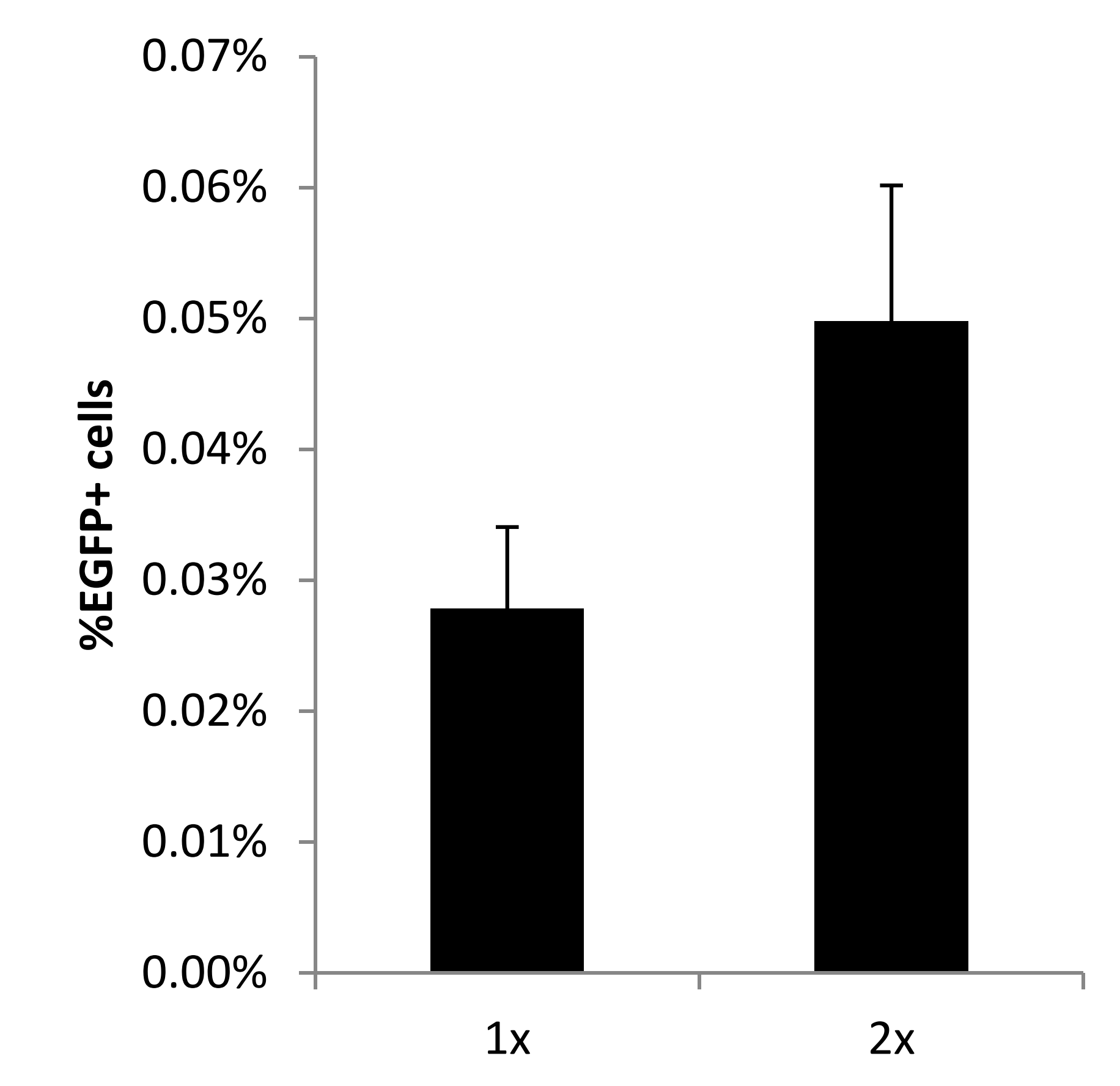

Supplement: Figure S4 — HeLa F5 cells transfected with F5-17 oligo, either 1x or 2x DNA:lipofectamine complexes, assayed for %EGFP+ cells eight days after transfection. (TIFF) [file pone.0036697.s004.tif]

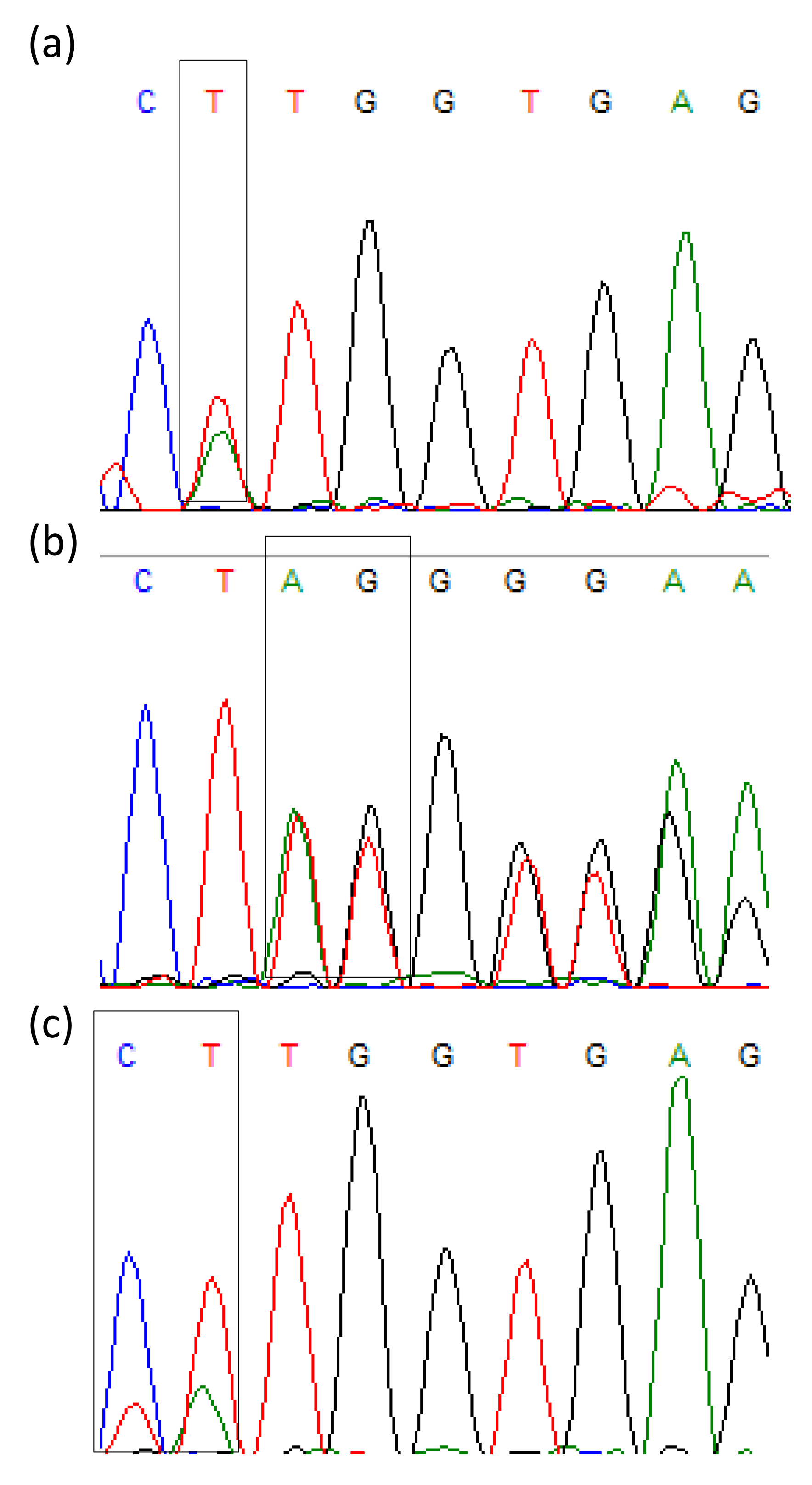

Supplement: Figure S5 — Sequencing corrected EGFP+ clonal cells. Single-cells were sorted into a 96-well plate 8 days post-transfection with (a) F5-17,(b)F5-34, (c)F5-35, grown for two weeks and then sequenced. Sequence spectrum overlap shows one of the two mEGFP copies was modified. (TIFF) [file pone.0036697.s005.tif]

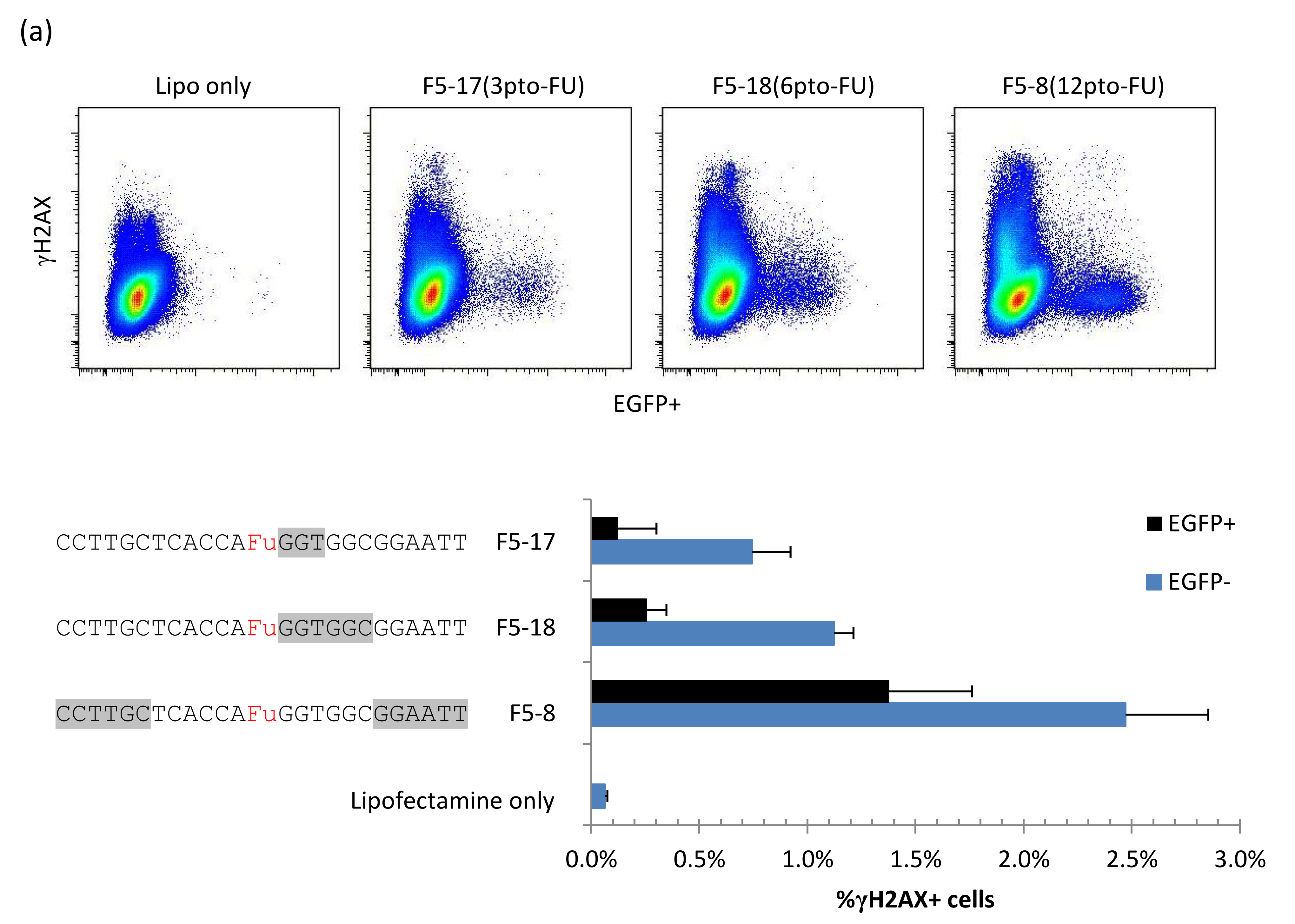

Supplement: Figure S6 — γ-H2AX phosphorylation staining. Cells were plated 500,000/well on a 6-well plate, then transfected with the corresponding oligos. After 36 hrs, cells were washed, fixed in 4%PFA, permeabilized with 10% saponin, then stained with Alexa Fluor 647 anti-H2A.X-Phosphorylated Antibody (1∶25, Biolegend) shaking for 20 mins at room temperature. (TIF) [file pone.0036697.s006.tif]
