# Supplementary material for: Stable Gene Targeting in Human Cells Using Single-Strand Oligonucleotides with Modified Bases
Source: PLoS One. 2012 May 14;7(5):e36697. doi: 10.1371/journal.pone.0036697 (PMC3351460; doi:10.1371/journal.pone.0036697)
Supplement: Table S1 — PCR primers sequences. (DOCX) [file pone.0036697.s007.docx]

**Table S1. PCR primers sequences**

| **PCR primers** | |
| --- | --- |
| **Name** | **Sequence** |
| N1 | TCAATGGGCGTGGATAGCGG |
| N3 | CGTTGTGGCTGTTGTAGTTG |
| EGFP(-)-specific forward primer | CGCTGAATTCCGCCACCt |
| EGFP(+)-specific forward primer | CGCTGAATTCCGCCACCa |
| EGFP Reverse primer | CAGGGTCAGCTTGCCGTAGG |
| **qPCR primers** | |
| **Name/Target** | **Sequence** |
| POLR2B Fwd(housekeeping) | GCGGATGAGGATATGCAATATGA |
| POLR2B Rvs | ACCAAGCCTTTCTCGTCAAAA |
| IL32 Fwd | ATGTGCTTCCCGAAGGTCCTC |
| IL32 Rvs | TCATTTTGAGGATTGGGGTTC |
| HLAB Fwd | CAGTTCGTGAGGTTCGACAG |
| HLAB Rvs | CAGCCGTACATGCTCTGGA |
| OAS3 Fwd | TCTGAGACTCACGTTTCCTGA |
| OAS3 Rvs | CACTGTTGAGGAGGGTAGAGTA |
