# Supplementary material for: Stable Gene Targeting in Human Cells Using Single-Strand Oligonucleotides with Modified Bases
Source: PLoS One. 2012 May 14;7(5):e36697. doi: 10.1371/journal.pone.0036697 (PMC3351460; doi:10.1371/journal.pone.0036697)
Supplement: Table S2 — shRNAs sequences. (DOCX) [file pone.0036697.s008.docx]

**Table S2. shRNAs sequences**

| Name | Target | Sequence |
| --- | --- | --- |
| TRCN0000040056 | MLH1 | CCGGCCAAGTGAAGAATATGGGAAACTCGAGTTTCCCATATTCTTCACTTGGTTTTTG |
| TRCN0000039670 | MSH2 | CCGGGCCTTGCTGAATAAGTGTAAACTCGAGTTTACACTTATTCAGCAAGGCTTTTTG |
| #1864 (Addgene) | Scramble | CCTAAGGTTAAGTCGCCCTCGCTCGAGCGAGGGCGACTTAACCTTAGG |
