# Supplementary material for: Stable Gene Targeting in Human Cells Using Single-Strand Oligonucleotides with Modified Bases
Source: PLoS One. 2012 May 14;7(5):e36697. doi: 10.1371/journal.pone.0036697 (PMC3351460; doi:10.1371/journal.pone.0036697)
Supplement: Table S3 — siRNA sequences. (DOCX) [file pone.0036697.s009.docx]

**Table S3. siRNA sequences**

| Name | Target site | Sequence |
| --- | --- | --- |
| SASI_WI_00000004 | 195 | CCGGCCAAGTGAAGAATATGGGAAACTCGAGTTTCCCATATTCTTCACTTGGTTTTTG |
| SASI_WI_00000009 | 2526 | CCGGGCCTTGCTGAATAAGTGTAAACTCGAGTTTACACTTATTCAGCAAGGCTTTTTG |
| DS Scrambled Neg (IDT) | Scramble |  |
