# Supplementary material for: Stable Gene Targeting in Human Cells Using Single-Strand Oligonucleotides with Modified Bases
Source: PLoS One. 2012 May 14;7(5):e36697. doi: 10.1371/journal.pone.0036697 (PMC3351460; doi:10.1371/journal.pone.0036697)
Supplement: Table S4 — Enriched GO categories. Top 400 genes based on p-value were analyzed with Toppgene, showing strong representation of viral process category. (DOCX) [file pone.0036697.s010.docx]

**Table S4. Enriched GO categories.**

| **GO ID** | **Name** | **P-value** |
| --- | --- | --- |
| GO:0006415 | translational termination | 5.45E-33 |
| GO:0016032 | **viral reproduction** | 1.26E-30 |
| GO:0019058 | **viral infectious cycle** | 1.43E-30 |
| GO:0022415 | **viral reproductive process** | 2.85E-30 |
| GO:0006414 | translational elongation | 2.98E-30 |
| GO:0034623 | cellular macromolecular complex disassembly | 8.34E-30 |
| GO:0019083 | **viral transcription** | 9.65E-30 |
| GO:0019080 | **viral genome expression** | 9.65E-30 |
| GO:0032984 | macromolecular complex disassembly | 2.01E-29 |
| GO:0031018 | endocrine pancreas development | 7.36E-28 |
| GO:0071845 | cellular component disassembly at cellular level | 1.56E-27 |
| GO:0022411 | cellular component disassembly | 2.92E-27 |
| GO:0043624 | cellular protein complex disassembly | 5.63E-27 |
| GO:0043241 | protein complex disassembly | 1.35E-26 |
| GO:0006412 | translation | 2.06E-26 |
| GO:0031016 | pancreas development | 4.57E-25 |
| GO:0034621 | cellular macromolecular complex subunit organization | 2.81E-21 |
| GO:0035270 | endocrine system development | 3.43E-21 |
| GO:0048610 | cellular process involved in reproduction | 1.10E-18 |
| GO:0071822 | protein complex subunit organization | 5.58E-16 |
| GO:0043933 | macromolecular complex subunit organization | 1.50E-15 |
| GO:0022414 | reproductive process | 3.49E-08 |
| GO:0000003 | reproduction | 4.03E-08 |
| GO:0006396 | RNA processing | 3.82E-07 |
| GO:0016071 | mRNA metabolic process | 5.80E-05 |
| GO:0007093 | mitotic cell cycle checkpoint | 1.44E-03 |
| GO:0008380 | RNA splicing | 2.34E-03 |
| GO:0042274 | ribosomal small subunit biogenesis | 2.93E-03 |
| GO:0012501 | programmed cell death | 7.88E-03 |
| GO:0006364 | rRNA processing | 1.01E-02 |
| GO:0006260 | DNA replication | 1.14E-02 |
| GO:0002576 | platelet degranulation | 1.25E-02 |
| GO:0016072 | rRNA metabolic process | 1.39E-02 |
| GO:0031575 | mitotic cell cycle G1/S transition checkpoint | 1.50E-02 |
| GO:0071779 | G1/S transition checkpoint | 1.50E-02 |
| GO:0006090 | pyruvate metabolic process | 1.50E-02 |
| GO:0046034 | ATP metabolic process | 1.82E-02 |
| GO:2000045 | regulation of G1/S transition of mitotic cell cycle | 1.97E-02 |
| GO:0042273 | ribosomal large subunit biogenesis | 2.02E-02 |
| GO:0072431 | signal transduction involved in mitotic cell cycle G1/S transition DNA damage checkpoint | 2.14E-02 |
| GO:0072395 | signal transduction involved in cell cycle checkpoint | 2.14E-02 |
| GO:0072413 | signal transduction involved in mitotic cell cycle checkpoint | 2.14E-02 |
| GO:0072404 | signal transduction involved in G1/S transition checkpoint | 2.14E-02 |
| GO:0072474 | signal transduction involved in mitotic cell cycle G1/S checkpoint | 2.14E-02 |
| GO:0006977 | DNA damage response, signal transduction by p53 class mediator resulting in cell cycle arrest | 2.14E-02 |
| GO:0072422 | signal transduction involved in DNA damage checkpoint | 2.14E-02 |
| GO:0072401 | signal transduction involved in DNA integrity checkpoint | 2.14E-02 |
| GO:0006096 | glycolysis | 2.38E-02 |
| GO:0000075 | cell cycle checkpoint | 3.43E-02 |
| GO:0071158 | positive regulation of cell cycle arrest | 3.59E-02 |
| GO:0006457 | protein folding | 3.59E-02 |
| GO:0006915 | apoptosis | 3.77E-02 |
| GO:0006397 | mRNA processing | 4.25E-02 |
| GO:0031571 | mitotic cell cycle G1/S transition DNA damage checkpoint | 4.79E-02 |
